# NMR crystallography of the high-pressure form of a multi-component active pharmaceutical ingredient

**DOI:** 10.1039/d6sc02621d

**Published:** 2026-07-29

**Authors:** Jiashan Mi, Amrit Venkatesh, Ivan Hung, Nathan Ho, Antonio G. DiPasquale, Joseph W. Lubach, Aaron J. Rossini

**Affiliations:** a Department of Chemistry, Iowa State University Ames IA 50010 USA arossini@iastate.edu +1 515-294-8952; b National High Magnetic Field Laboratory, Florida State University Tallahassee FL 32310 USA; c Department of Chemistry, University of Virginia Charlottesville VA 22904 USA; d Genentech, Inc., Department of Small Molecule Pharmaceutical Sciences 1 DNA Way South San Francisco CA 94080 USA josephwl@gene.com dipasqua@gene.com +1 650-225-5072 +1 650-491-7170

## Abstract

Pressure and mechanical forces during pharmaceutical manufacturing can induce solid-phase transformations of active pharmaceutical ingredients (APIs), posing risks to product quality. Here, we report the structural characterization of a metastable high-pressure polymorph of the multi-component GDC-0022 tosylate salt formed under applied pressure of 250 MPa. The crystal structure of the high-pressure polymorph is determined with a combination of ^1^H, ^14^N and ^19^F solid-state NMR (SSNMR) spectroscopy and crystal structure prediction (CSP). Distinct ^1^H and ^19^F SSNMR signals, longitudinal relaxation time (*T*_1_) measurements, and 2D ^19^F spin-diffusion spectra confirm that the high- and low-pressure forms coexist as separate crystalline domains. 2D ^19^F{^1^H} hetero-nuclear correlation (HETCOR) and ^1^H double-quantum single-quantum (DQ-SQ) NMR spectra resolve key ^1^H NMR signals of each phase, while ^1^H{^14^N} *J*-HMQC experiments establish that the high-pressure form retains salt character, with one nitrogen atom remaining protonated. CSP combined with DFT GIPAW chemical shift calculations identifies a high-density polymorph consistent with the experimental ^1^H and ^19^F chemical shifts. These results demonstrate that NMR crystallography can deconvolute complex polymorphic mixtures and provide a practical framework for managing pressure-induced phase transitions in drug manufacturing.

## Abstract 2

Crystal structure prediction and ^1^H and ^19^F NMR crystallography were used to solve the structure of a high-pressure solid drug form.

## Graphical abstract

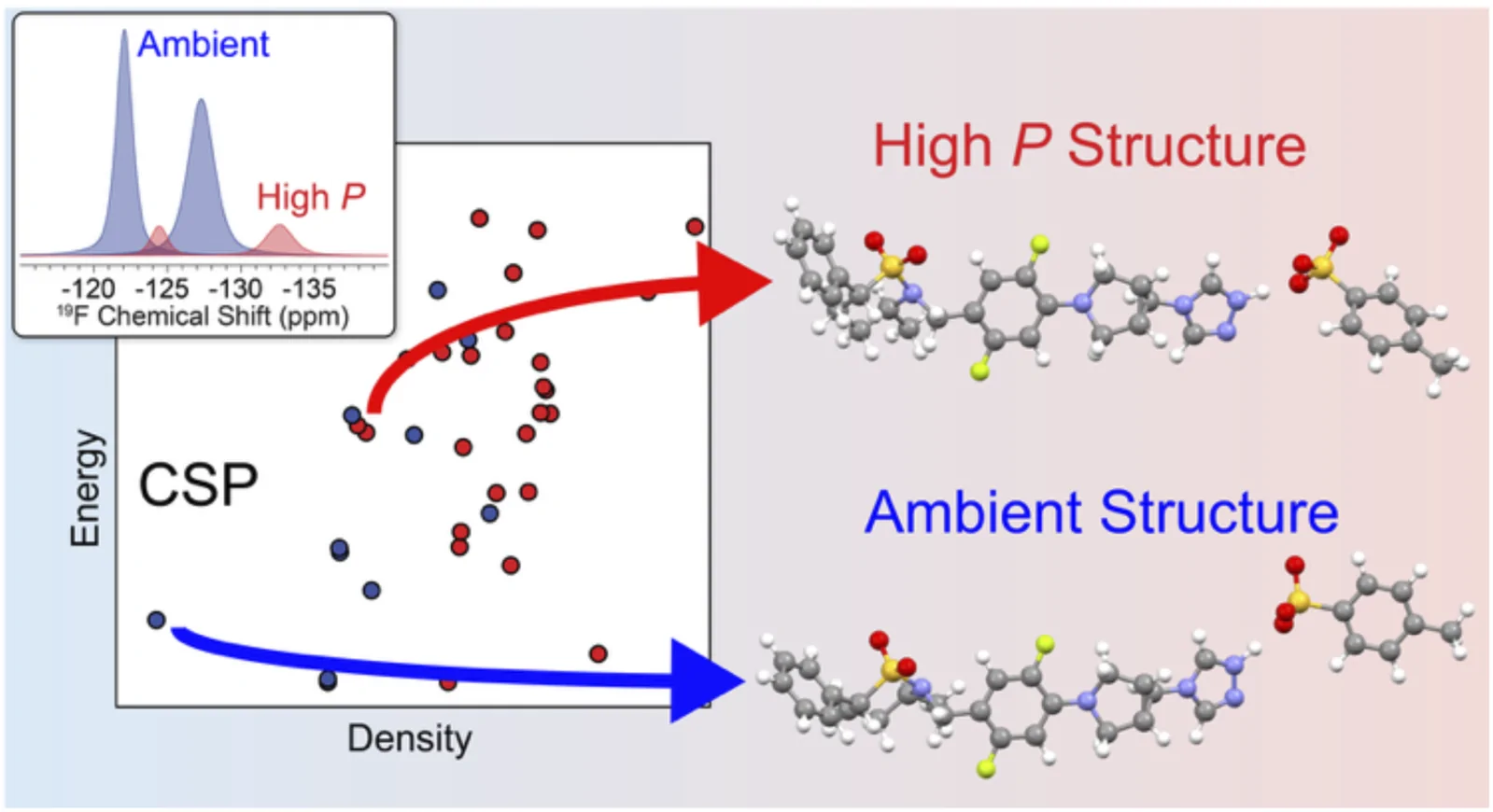

## 1 Introduction

The solid-state form of an active pharmaceutical ingredient (API) is a critical determinant of its clinical performance. Polymorphism, the ability of a compound to exist in multiple crystalline forms, can profoundly influence key physicochemical properties, including solubility, dissolution rate, chemical stability, and bioavailability.^1^ The unforeseen appearance of a more stable, and possibly less soluble, polymorph during late-stage development or post-launch can have severe financial and therapeutic consequences, as famously illustrated by the case of ritonavir,^2–4^ where the emergence of a new crystal form compromised the drug's bioavailability and forced a market withdrawal. Consequently, exhaustive screening and characterization of all accessible solid forms are mandatory, risk-mitigating activities in modern pharmaceutical development.^5^ Manufacturing processes such as grinding, milling, and tablet compaction routinely subject APIs to significant mechanical stress. These operations can generate high localized pressures^6–8^ sufficient to induce solid-state phase transformations, potentially converting the desired therapeutic crystal form into a different, uncharacterized polymorph.

Concurrently, the deliberate application of high pressure is emerging as a valuable tool for systematically exploring a compound's crystal energy landscape to discover novel solid forms.^9^ The thermodynamic stability of a given polymorphic phase is governed by the molar Gibbs free energy (*G*_m_), defined as *G*_m_ = *H*_m_ − *TS*_m_ = *U*_m_ + *PV*_m_ − *TS*_m_, where *H*_m_ is the molar enthalpy, *U*_m_ is the molar internal energy, *P* is pressure, *V*_m_ is molar volume, *T* is temperature and *S*_m_ is molar entropy. Under standard ambient conditions, the pressure-volume term (*PV*_m_) makes a negligible contribution to the relative free energy or relative enthalpy differences between polymorphs. However, because the pressure dependence of the Gibbs free energy is directly related to the molar volume at constant temperature, 
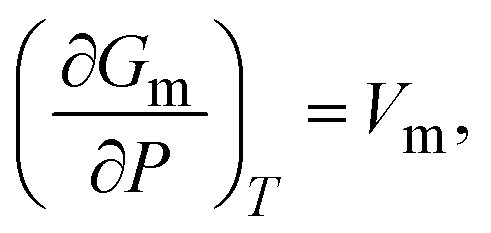
 applying pressures on the order of gigapascals (GPa) dramatically increases the contribution of the *PV*_m_ term.^7,10^ For example, assuming molar volume differences of 30 cm^3^ mol^−1^ between two different solid forms, under a pressure of 1 GPa, the *PV*_m_ term will lead to a 30 kJ mol^−1^ change in relative free energy of the two different solid forms (assuming there are no changes in molar volume of each form during pressurization). The thermodynamic driving force of higher pressure favors denser packing arrangements that minimize volume, stabilizing structures that may be energetically unfavorable or inaccessible at ambient pressure. For instance, the well-studied analgesic paracetamol was found to exhibit two novel high-pressure polymorphs, forms IV and V, that appear at pressures exceeding 8 GPa and 11 GPa, respectively.^11^ Similarly, glycine displays a complex cascade of pressure-induced phase transitions, transforming to the δ-form at 0.8 GPa and the ε-form at 4.3 GPa, with a distinct ζ-polymorph appearing solely upon decompression.^12,13^ Investigations into xylazine hydrochloride have revealed that high pressure can differentially stabilize solvated forms over pure polymorphs, with unsolvated form *Z* being favored only within a narrow window below 0.1 GPa.^14^ To put these pressures into the context of pharmaceutical manufacturing, maximum tablet compaction pressures are typically on the order of 0.25 GPa, though usually less pressure is required to achieve the desired tablet solid fraction.

Complementing these experimental efforts, computational crystal structure prediction (CSP) has proven instrumental in guiding the discovery of solid drug phases.^4,15–22^ A landmark study on dalcetrapib demonstrated that CSP could successfully predict a thermodynamically competitive polymorph, which was subsequently isolated *via* high-pressure crystallization, thereby effectively de-risking the solid-form landscape.^9^ Recently, CSP was applied to 5-methyl-2-[(2-nitrophenyl)amino]-3-thiophenecarbonitrile (ROY), the current record holder for the most polymorphic organic molecule, with CSP predicting the existence of a new polymorph stable only above 10 GPa of pressure.^23^ These findings suggests that even for exhaustively studied systems like ROY, the high-pressure regime remains a fertile ground for the identification of new polymorphs.

A significant analytical challenge lies in the structural characterization of pressure-induced phases. Solid drug forms are often obtained as microcrystalline powders that are unsuitable for single-crystal X-ray diffraction, and high-pressure drug forms will likely be obtained as a minority component within a mixture of the initial, ambient-pressure form.^24,25^ Solid-state nuclear magnetic resonance (SSNMR) spectroscopy is a powerful, non-destructive technique uniquely suited for characterizing complex, heterogeneous solid systems.^26,27^ SSNMR spectroscopy has been shown to be applicable to both formulated pharmaceuticals^28–32^ and mixtures of solid drug forms.^33^ The chemical shifts observed in SSNMR spectra are sensitive to the subtle differences in crystal packing and conformation observed in different polymorphs.^34^ The integration of SSNMR spectroscopy with CSP and density functional theory (DFT) calculations of NMR parameters has given rise to the field of NMR crystallography.^25,35–37^ Most NMR crystallography protocols rely upon the comparison of experimental and calculated ^1^H and ^13^C NMR chemical shifts to sort the CSP structures.^22,38,39^ Alternatively, measurement of ^1^H–^1^H, ^1^H–^13^C or ^13^C–^13^C internuclear proximities *via* dipole-based two dimensional (2D) SSNMR experiments has also proven to be a powerful method for structure determination.^40–43^ The field has also expanded in the past few years to exploit other nuclei, including quadrupolar nuclei like ^35^Cl and ^43^Ca, to provide structural constraints.^44–46^ Due to the prevalence of fluorine within pharmaceuticals, ^19^F SSNMR spectroscopy has attracted considerable attention for studying solid APIs, as the high gyromagnetic ratio and 100% natural abundance of ^19^F provide high sensitivity, which is further enhanced by the availability of fast magic angle spinning (MAS) probes.^47–50^

This study demonstrates a comprehensive NMR crystallography workflow to deconvolute a solid mixture consisting of ambient and high-pressure forms of the multi-component API, GDC-0022 tosylate salt. We show that multi-nuclear (^1^H, ^14^N, ^19^F) SSNMR experiments, particularly two-dimensional correlation techniques, can be used to spectroscopically isolate the NMR signatures of the major ambient-pressure phase and the minority metastable high-pressure phase. These phase pure NMR chemical shifts serve as precise experimental filters to interrogate a landscape of computationally predicted structures, enabling the crystal structure of the minority high-pressure phase to be determined. This integrated approach provides a robust framework for the structural elucidation of pressure-induced polymorphs, offering a valuable tool for understanding and controlling phase transitions that may occur during pharmaceutical manufacturing.

## 2 Experimental methods

### 2.1 Sample preparation

GDC-0022 tosylate salt^51^ was obtained as a crystalline solid (ambient phase) by Genentech, Inc. To induce high-pressure polymorphic transformations, powdered samples were compressed using a STYL’One Nano compaction simulator (Medelpharm SAS, Beynost, France). Using the built-in extended dwell time profile, the samples were held at pressures of approximately 250 MPa for a duration of 3 seconds. The weight of each tablet was 350 ± 10 mg. The punches and die used in this study were round, flat face with a diameter of 10 mm. For initial pressure screening, samples were held at pressures of 50, 150, and 250 MPa for a duration of 3 s. Following depressurization, the samples were visually inspected for color changes and gently ground into loose powders that could be packed into NMR rotors for analysis. To achieve higher conversion for further characterization, approximately 100 mg of a powdered sample, which had been previously pre-compressed at 250 MPa using the procedure described above, was loaded into a die set. The assembly consisted of 8 mm standard convex TDP tooling (LFA Tablet Presses, Fort Worth, TX) housed within a custom-fabricated aluminum alignment sleeve. The sample was then compressed using a Carver Laboratory Press Model B (Fred S. Carver Inc., Summit, NJ) at a pressure of approximately 1800 MPa and held for 30 minutes to ensure maximum densification.

### 2.2 Powder X-ray diffraction (PXRD)

Finely ground powders were studied by powder X-ray diffraction (PXRD) using a Rigaku Miniflex 600 diffractometer with a Cu-Kα radiation and Ni-Kβ filter (Fig. S1). A thin layer of uniform grease was used for sample preparation.

### 2.3 Solid-state NMR spectroscopy experiments

SSNMR experiments were conducted across multiple magnetic fields (9.4 T, 11.7 T, 14.1 T, and 18.8 T) at ambient temperature. Chemical shifts were referenced to neat TMS (^1^H), CFCl_3_ (^19^F), and nitromethane (^14^N). Comprehensive instrumental parameters, including MAS frequencies, pulse lengths, continuous-wave/decoupling RF field strengths, and acquisition delays for all experiments, are detailed in the SI. A schematic illustration of all pulse sequences is given in Fig. S2. While ^13^C and ^15^N SSNMR spectroscopy are commonly applied to study polymorphs, their application here is limited by sensitivity and resolution constraints for the minor high-pressure phase. The ^15^N natural abundance (0.37%) typically requires isotope enrichment DNP enhancement, or extremely long signal averaging times (as previously demonstrated for pharmaceutical salts; see Zhao *et al.*, *Cryst. Growth Des.* 2018).^52^ Furthermore, the ^13^C CPMAS spectrum of the 250 MPa mixture exhibits severe spectral overlap between the ambient and high-pressure components, precluding unambiguous resonance assignment for the minor phase (Fig. S3). Therefore, ^1^H and ^19^F were targeted for NMR spectroscopy due to their 100% natural abundance, high gyromagnetic ratios, and exceptional sensitivity to local packing environments, which are essential for deconvoluting polymorphic mixtures. The experimental methods were selected to address specific structural questions regarding the high-pressure polymorph.

#### 2.3.1 Phase identification and quantification

1D ^19^F spin echo and ^1^H → ^19^F CPMAS experiments were used to identify the presence of the new high-pressure phase and quantify its relative abundance.

#### 2.3.2 Phase segregation and domain sizes

^1^H and ^19^F spin-lattice relaxation (*T*_1_) times were measured *via* saturation recovery to evaluate phase segregation. These experiments were complemented by 2D ^19^F → ^19^F spin diffusion and 2D spin diffusion ^1^H → ^19^F CP-HETCOR experiments to probe spatial proximities and confirm the existence of distinct crystalline domains.

#### 2.3.3 Chemical shift resolution

To resolve the specific ^1^H chemical shifts for the bulk ambient and high-pressure phases, 2D ^1^H → ^19^F CP HETCOR experiments were performed. A ^1^H spin diffusion period was incorporated to equilibrate magnetization across the proton network. Additionally, a dipole-based 2D ^1^H double-quantum single-quantum (DQ-SQ) correlation NMR spectrum of the compressed sample was obtained with a 50 kHz MAS frequency and an 18.8 T magnetic field. The 2D ^1^H DQ-SQ NMR spectrum shows correlations for ^1^H NMR signals that arise from ^1^H atoms that are within a few Angstroms of one another. This spectrum allows additional ^1^H chemical shifts to be resolved by correlating the resolved ^1^H NMR signals from the NH groups of each phase to ^1^H NMR signals from the CH groups of the triazole rings.

#### 2.3.4 Protonation state and bonding

High-field (18.8 T) fast MAS (50 kHz) 2D ^1^H{^14^N} *J*-HMQC experiments were utilized to probe the NH bonding environment and determine whether the high-pressure solid form remained a salt.

### 2.4 CSP using GRACE

Crystal structure prediction was performed using the Generation Ranking and Characterization Engine (GRACE) version 2.8.54 from Avant-garde Materials Simulation Deutschland GmbH. Two separate CSP runs were performed with pressures of 0 MPa and 250 MPa. Tailor made forcefields were generated for the GDC-0022 tosylate salt and backfitted against crystal structures generated by GRACE and optimized by plane-wave DFT. Theoretical crystal structures were then generated (*Z*′ was limited to 1 for the 250 MPa structure search due to computational constraints) using the backfitted forcefields for 21 chiral space groups with the highest probability for molecules not on special positions. Predicted crystal structures were optimized by plane-wave DFT to give lattice enthalpies (*H* = *U* + *PV*) at the applied pressure. Gibbs free energies were subsequently computed for the subset of lowest-enthalpy candidate structures by incorporating entropic contributions obtained from phonon calculations within the GRACE workflow, yielding Gibbs free energies (*G* = *H* − *TS*). The *TS* entropic contributions are generally small relative to the enthalpic term *H* = *U* + *PV*. Relative Gibbs free energies are reported here to maintain consistency with the standard GRACE workflow output. Rankings of the CSP structures by relative molar Gibbs free energy and relative molar enthalpies are shown in Fig. S4.

### 2.5 Plane-wave DFT calculations

All calculations were performed using plane-wave pseudopotential periodic DFT as implemented in the CASTEP version 2018 code.^53^ All calculations used the Perdew–Burke–Ernzerhof generalized gradient approximation (PBE-GGA) functional^54^ with ultrasoft pseudopotentials generated on-the-fly.^39^ The Tkatchenko–Scheffler (TS) dispersion correction scheme was used in all calculations.^55^ The wave functions were expanded using a plane-wave basis set with a kinetic energy cutoff of 630 eV that produces converged results for both the geometry optimization and the calculation of NMR parameters. NMR shielding values were calculated using the GIPAW method^56^ as implemented in the CASTEP code. The integrals were calculated over the Brillouin zone and were performed using a Monkhorst–Pack grid with a *k*-point spacing of 0.07 Å^−1^. Relativistic effects on energies were incorporated using the zeroth-order regular approximation (ZORA) pseudopotential formalism.^57^ The XRD crystal structure of GDC-0022 tosylate salt (CCDC# 1822443) was used as the initial structure. Prior to GIPAW calculation of chemical shifts the positions of all atoms were optimized, while keeping the unit cell dimensions from the crystal structure fixed. DFT-calculated ^19^F isotropic magnetic shieldings (*σ*_iso_) were converted to isotropic chemical shifts (*δ*_iso_) through a chemical shielding to chemical shift calibration plot constructed from previously reported compounds with known crystal structures and measured solid-state ^19^F chemical shifts (Fig. S5 and Table S1). ^19^F chemical shifts are recognized to be particularly challenging to predict accurately by DFT methods, owing to the high sensitivity of fluorine shielding to local electronic environment, molecular conformation, and crystal packing.^25,47^ Previous benchmarking studies of DFT-based ^19^F NMR shift predictions have reported RMSE of approximately 3.6 ppm for solution-state calculations^58^ and ∼4–5 ppm for periodic GIPAW calculations of organic solids.^25,59^ We have performed PBE-GIPAW ^19^F magnetic shielding calculations (*σ*_iso_) for 16 distinct ^19^F sites across 9 model solid compounds with known ^19^F chemical shifts (Table S1). Fitting the calculated magnetic shieldings to the experimental ^19^F chemical shifts yielded the relationship *δ*_iso_ = (−0.9069) (*σ*_iso_) + 121.6450 ppm (Fig. S5). These calibration parameters represent the mean slope and intercept derived from leave-one-out cross-validation (LOOCV). From this calibration set, the RMSD of the calculated ^19^F chemical shift was determined to be 2.23 ppm. To establish a rigorous statistical threshold for structure selection using only two ^19^F chemical shifts, a standard deviation (*σ*) of 0.72 ppm was determined by evaluating the pairwise RMSD across all *C*(16,2) = 120 unique chemical shift pairs of the calibration set. ^1^H chemical shifts (*δ*_iso_) were determined using the PBE-GIPAW shielding-shift calibration curve parameters (*δ*_iso_ = (−0.8731(*σ*_iso_) + 27.20 ppm)) previously published by Beran and co-workers, who used similar *k*-point spacings, but a higher energy cutoff (*ca.* 1088 eV) in their calibration data.^60^ The experimental ^1^H chemical shifts in this study were referenced to adamantane at 1.82 ppm. To ensure consistency with the established ^1^H calibration curve which referenced adamantane to 1.87 ppm, a systematic correction offset of +0.05 ppm was applied to our experimental shifts prior to calculating the absolute prediction errors. To ensure the established ^1^H calibration curve is directly applicable to our specific computational setup, we evaluated its predictive performance on three polymorphs/solvates of theophylline (Form I, Form II, and Monohydrate) with known ^1^H chemical shifts.^32^ We also compared the calculated and experimental ^1^H chemical shifts for l-Histidine·HCl·H_2_O. Using the Beran shielding-shift calibration curve results in an RMSD of 0.30 ppm for the calculated ^1^H chemical shifts, confirming that their calibration curve is valid and compatible with the calculations performed here (Table S2). From the previously established ^1^H shielding calibration dataset of Beran and co-wokers, the cross-validation RMSD of calculated ^1^H chemical shifts was determined to be 0.47 ppm. A standard deviation of 0.16 ppm was determined on the RMSD by evaluating the RMSD across all *C*(80,3) = 82 160 unique chemical shift triplets present in the calibration data set. This standard deviation was used as the uncertainty on the RMSD cutoff. Finally, to evaluate candidate structures, we also calculated reduced *χ*^2^ metrics using the methods of Mueller, as described below. Analysis of reduced chi-square metrics was used to evaluate candidate structures and assign them probabilities of being the correct structure (see SI and main text below).

## 3 Results and discussion

### 3.1 ^1^H and ^19^F solid-state NMR spectra reveal a pressure-induced phase transformation

**Fig. 1:**
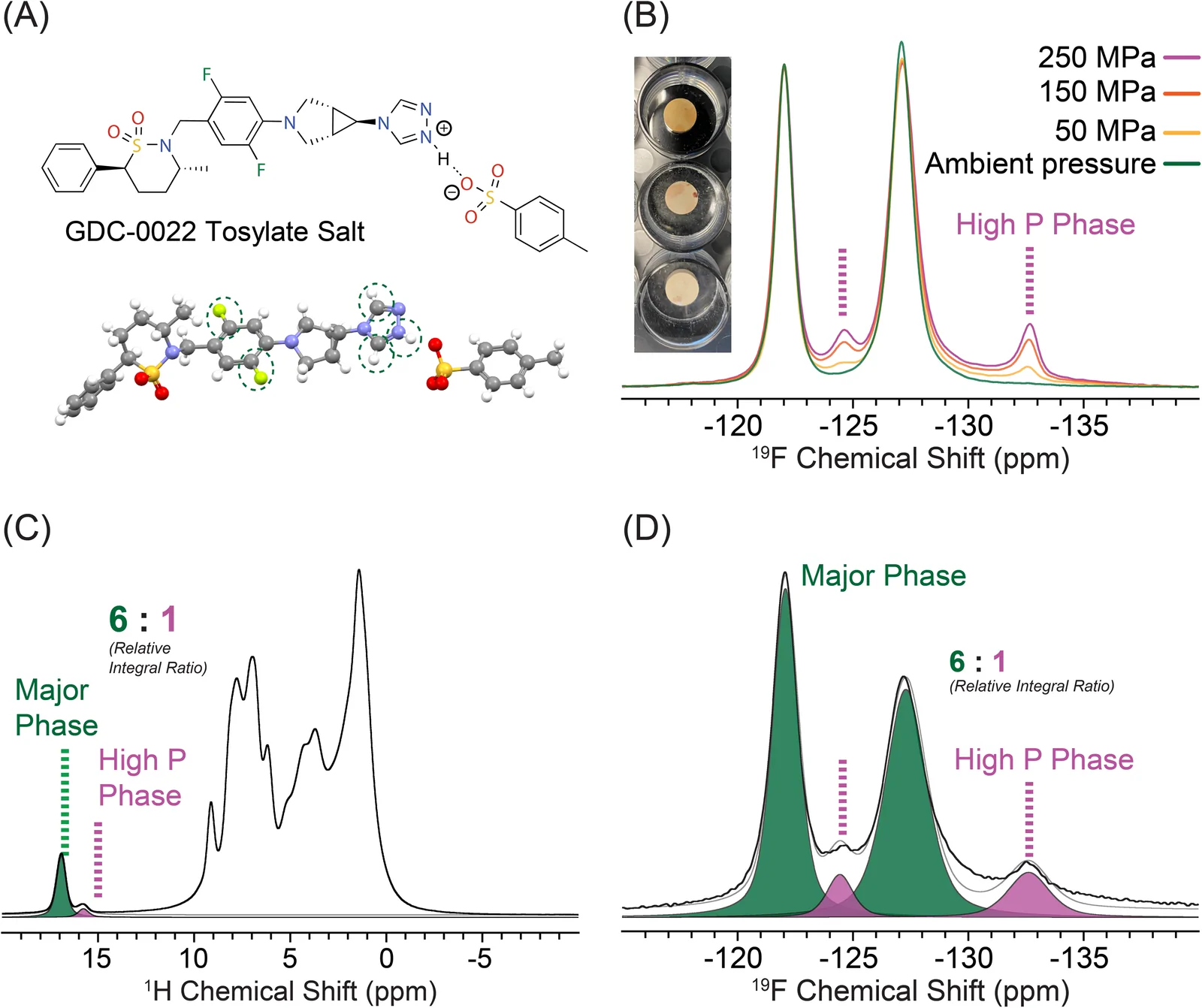
(A) Line structure and single-crystal X-ray structure of GDC-0022 tosylate salt ambient pressure phase. The ^19^F atoms, ammonium ^1^H atom and triazole CH ^1^H atoms are circled on the crystal structure. The chemical shift of these atoms were used for NMR crystallography. (B) ^1^H → ^19^F{^1^H} CPMAS spectra of GDC-0022 tosylate salt, showing the presence of new signals attributed to a high-pressure polymorph that forms after holding the drug at the indicated pressures for 3 seconds. The inset shows photographs of the pellets compressed at 250 MPa, 150 MPa, and 50 MPa (from top to bottom). (C) ^1^H and (D) ^19^F spin echo SSNMR spectra of the GDC-0022 tosylate salt pressurized at 250 MPa. New signals attributed to the high-pressure polymorph are indicated. All NMR experiments were performed at ambient pressure.

The effect of high-pressure mechanical stress on the solid form of GDC-0022 tosylate salt was first investigated using ^19^F solid-state NMR to probe for potential polymorphic transformations. Fig. 1A shows the molecular structure and single-crystal X-ray diffraction structure of the tosylic acid salt of GDC-0022 obtained under ambient pressure. For samples handled under ambient pressure two ^19^F resonances (green peaks) are observed, consistent with the two crystallographically distinct fluorine atoms present in the known single-crystal X-ray structure of this phase.

The pressurized samples were made by holding the powdered solid for 3 s at pressures of 50 MPa, 150 MPa or 250 MPa. After the pressure was released, the powders exhibit a darkening coloration, with the darkening correlating with the applied pressure. The change in color suggests the possible presence of a new high-pressure solid form, which remains metastable at ambient pressure. The ^1^H → ^19^F{^1^H} CPMAS spectra recorded at 11.7 T in Fig. 1B show a distinct pressure-dependent change, which the emergence of two distinct ^19^F NMR signals in pressurized samples. The new ^19^F NMR signals appear at chemical shifts of −124.5 ppm and −132.5 ppm. The intensity of these new signals correlates with the applied pressure, suggesting the formation of a new metastable high-pressure polymorph (peaks labelled High *P*). ^1^H SSNMR spectra also provide spectroscopic fingerprints of this new phase. As shown in Fig. 1C, the ^1^H SSNMR spectrum of the 250 MPa sample recorded with a 50 kHz MAS frequency reveals a new shoulder at approximately 15.8 ppm, at a lower chemical shift than the main N–H proton resonance of the ambient phase which appears at 16.9 ppm. The assignments for the ambient-pressure phase within the 250 MPa mixture were established by cross-referencing the NMR spectra with the extensively characterized pure ambient form of GDC-0022 tosylate, whose ^1^H, ^13^C, and ^14^N solid-state NMR spectra have been previously published.^52,61^ Specifically, the ambient phase contributions in the deconvoluted ^1^H spectrum (Fig. 1C) were assigned based on the previously reported ^1^H chemical shifts, the ^19^F chemical shifts observed in ambient samples, and those observed in the 2D ^1^H → ^19^F HETCOR spectra shown below. ^13^C CPMAS NMR spectra of a pressurized sample show the emergence of several new peaks, consistent with the formation of a new phase (Fig. S3). ^15^N CPMAS NMR spectra were not acquired, because approximately a week or more of signal averaging would likely be required to obtain high enough quality spectra to see signals from the minority high-pressure phase. However, as described below, we have performed ^1^H-detected ^14^N solid-state NMR experiments to probe the structure of the high-pressure phase.

A quantitative ^19^F spin echo NMR spectrum of the sample pressurized at 250 MPa was obtained with a 10 kHz MAS frequency (without application of ^1^H decoupling). To guarantee the quantitative accuracy of the integrated peak areas, extended recycle delays of 18.0 s and 30.0 s were employed for the ^1^H and ^19^F SSNMR experiments, respectively, ensuring nearly complete magnetization recovery. By fitting and integrating the ^1^H and ^19^F NMR spectra, the relative ratio of the ambient phase to the new high-pressure phase is determined to be approximately 6 : 1 from both the ^1^H and ^19^F NMR spectra (Fig. 1C and D). Therefore, after pressurization at 250 MPa, approximately 14% of the GDC-0022 tosylate remains as the metastable high *P* phase.

As an additional test, we attempted to increase the fraction of the high-pressure phase by using a Carver press to compress a sample at approximately 1800 MPa and held the sample at this pressure for 30 minutes. While this pressure clearly exceeds those typically experienced in pharmaceutical manufacturing, the experiment was utilized purely as an exploratory thermodynamic lever to further push the system towards the high *P* phase. The ^19^F NMR spectrum of this sample suggests that 23% of the sample remained as the high-pressure phase after decompression (Fig. S6). The partial conversion to the high *P* form suggests that the applied pressure is sufficient to initiate but not fully complete the phase transition. It is also possible that a majority of the sample undergoes a phase transition to the high-pressure form, however, after the pressure is released, only a fraction of the sample remains as the high-pressure phase. Additional experiments are required to test this hypothesis.

To evaluate the long-term stability of the metastable high-pressure phase after depressurization, we monitored the ^19^F NMR spectra of the sample pressurized at 250 MPa. ^19^F spin echo NMR spectra acquired 18 months apart show no significant changes in the relative intensities of the signals associated with the two phases (Fig. S7), confirming that conversion back to the ambient phase must be kinetically hindered.

### 3.2 Evidence for phase segregation from ^1^H and ^19^F saturation recovery and spin diffusion experiments

**Fig. 2:**
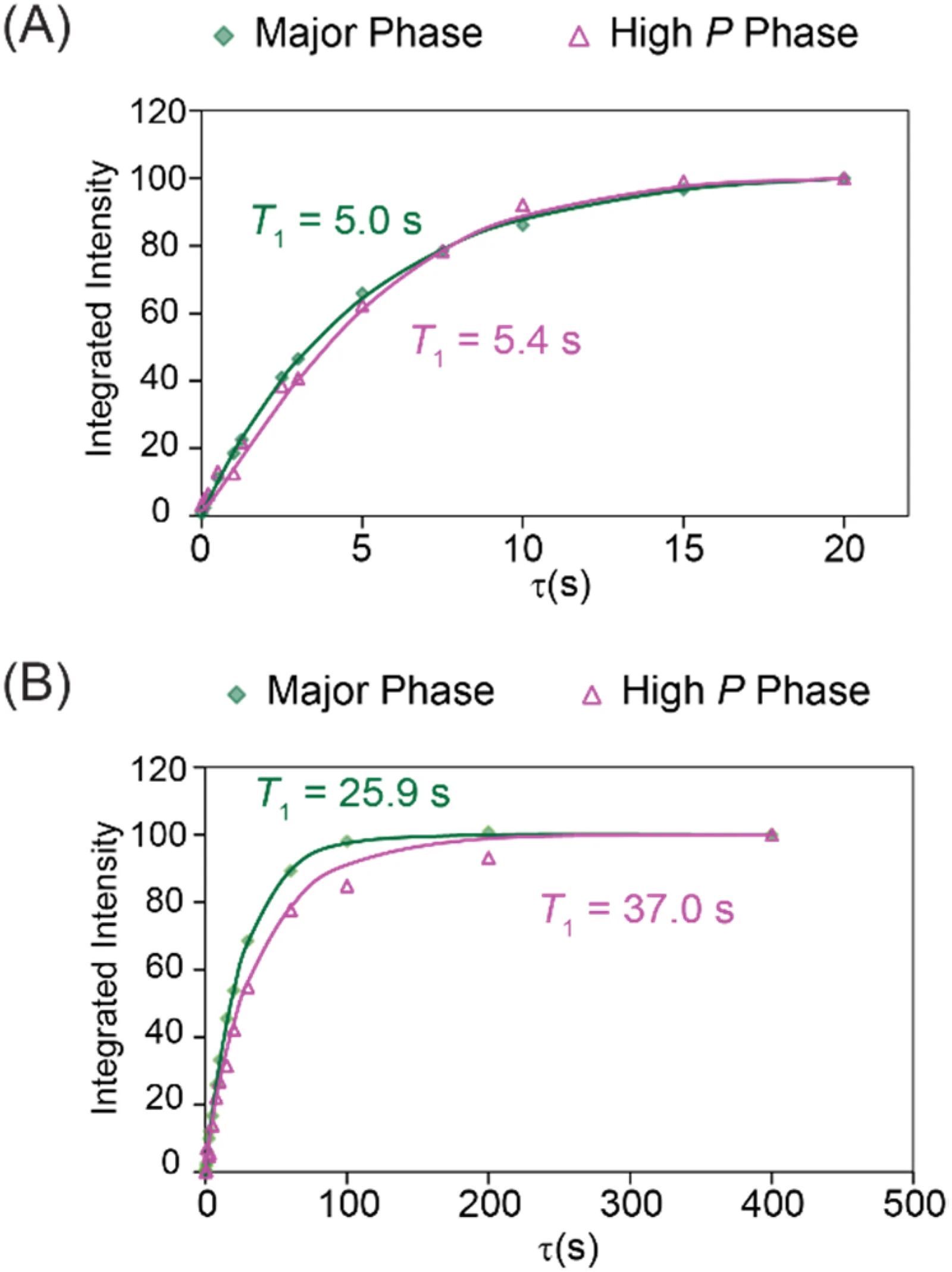
(A) ^1^H and (B) ^19^F spin-lattice relaxation (*T*_1_) saturation recover curves for the major phase (green) and the high-pressure phase (purple). Saturation recovery curves were fit using a non-linear least-squares MATLAB algorithm to a general exponential model of the form *I*(τ) = *I*_offset_ + *I*_eq_[1 − exp(–*τ*/*T*_1_)^*β*^], where *I*(*τ*) is the signal intensity at recovery time *τ*, *I*_offset_ is the baseline offset representing the residual unsaturated signal, *I*_eq_ is the equilibrium signal intensity, *T*_1_ is the spin-lattice relaxation time, and *β* is the stretch exponent accounting for the distribution of relaxation times due to sample heterogeneity. The pulse sequences used for *T*_1_ measurements are shown in Fig. S2E (^1^H → ^19^F CP saturation recovery) and Fig. S2D (^19^F saturation recovery). The distinct ^19^F *T*_1_ values (25.9 ± 0.5 s and 37.0 ± 2.3 s for the major and high-pressure phases, respectively) differ by 4.7 standard errors, with uncertainties propagated from 95% confidence intervals from the MATLAB fits, indicating that the two polymorphs exist in segregated crystalline domains.

**Fig. 3:**
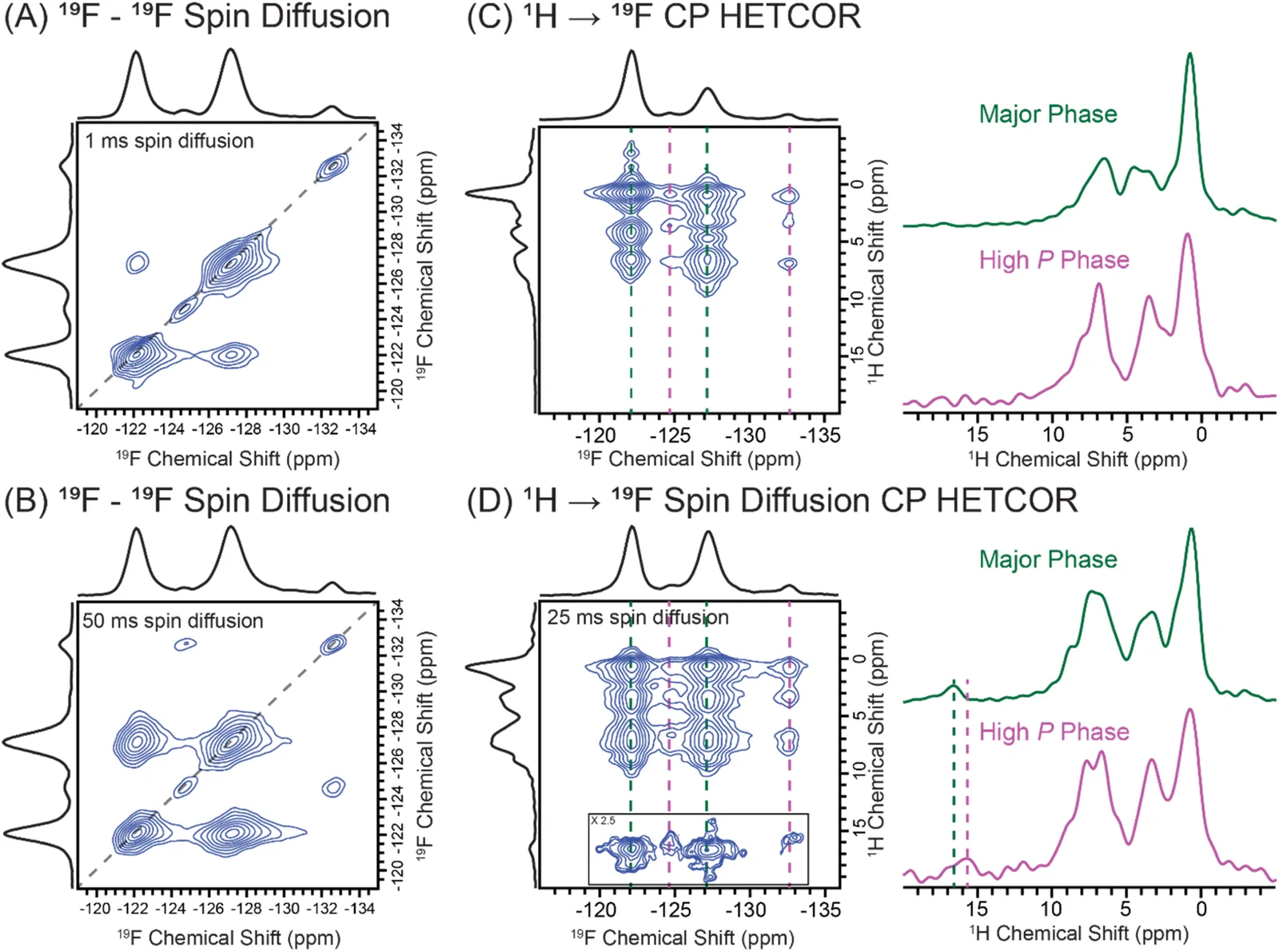
MAS 2D ^19^F spin diffusion NMR spectra recorded with a (A) 1 ms or (B) 50 ms spin diffusion time. No correlations are observed between the two phases, suggesting they are segregated. (C) ^1^H → ^19^F 2D HETCOR spectrum recorded with a contact time of 0.6 ms. (D) ^1^H → ^19^F 2D HETCOR with a 25 ms ^1^H spin diffusion period before the CP step to enable all ^1^H chemical shifts of the two phases to be observed. The columns indicated on the 2D HETCOR NMR spectra were summed to obtain the ^1^H NMR spectra of the major phase and high-P phase shown to the right of the HETCOR NMR spectra. *e*DUMBO_1–22_ homonuclear decoupling was applied during the *t*_1_-evolution period to enhance the resolution of the ^1^H NMR spectrum. All NMR experiments were performed with a MAS frequency of 10 kHz and a magnetic field of 14.1 T.

To probe the spatial relationship between the ambient pressure and high-pressure polymorphic forms, a series of SSNMR experiments sensitive to long-range internuclear distances were performed on the sample compressed at 250 MPa pressure. Longitudinal relaxation time (*T*_1_) measurements were performed on the 250 MPa sample to evaluate phase coexistence and assess the spatial separation of the ambient and high-pressure domains. Measurement of *T*_1_ provides a robust method to probe the domain sizes in heterogeneous solids.^33,62–64^ The ^1^H and ^19^F magnetization in a rigid lattice equilibrates spatially through spin diffusion.^65^ If the two phases are mixed intimately on a nanometer scale, the magnetization would average out, resulting in a single averaged *T*_1_ value for both components.^66^ Conversely, distinct *T*_1_ values imply that the domain sizes exceed the spin diffusion length scale over the timescale of the relaxation. As shown in Fig. 2, the ^1^H *T*_1_ and ^19^F *T*_1_ relaxation times were measured independently for the signals corresponding to the major and high-pressure phases. *T*_1_ relaxation times were measured with a MAS frequency of 10 kHz MAS frequency to ensure that ^1^H and ^19^F spin diffusion rates were maximized. The ^1^H *T*_1_ values were determined using a ^1^H → ^19^F CP saturation recovery sequence, where the ^19^F signal detection was used to enhance resolution of the two phases. The data reveal clearly distinct relaxation behaviors for the two forms. The ^1^H *T*_1_ values, measured *via* the ^1^H → ^19^F CP saturation recovery sequence, are 5.0 ± 0.1 s and 5.4 ± 0.3 s for the major and high-pressure phases, respectively. These values differ by approximately 1 standard error and are therefore not statistically distinguishable. By contrast, the ^19^F *T*_1_ values are 25.9 ± 0.5 s and 37.0 ± 2.3 s for the major and high-pressure phases, respectively, a difference of 11.1 s (4.7 standard errors), which is statistically significant. The clearly distinct ^19^F *T*_1_ values, together with the absence of ^19^F–^19^F spin diffusion cross-peaks between the two phases (Fig. 3A and B, see below), and the observation of distinct ^1^H solid-state NMR spectra in the ^1^H spin diffusion 1H → ^19^F CP-HETCOR spectrum (Fig. 3C and D, see below) provides additional evidence that the two polymorphs exist as physically segregated crystalline domains.

Theoretical models of spin diffusion suggest that for protons in organic solids, magnetization can diffuse over distances of tens of nanometers within a few seconds. This length scale is estimated using the relationship 
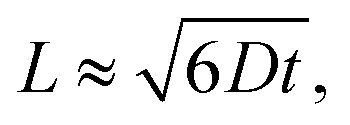
 where *L* is the diffusion length, *D* is the spin diffusion coefficient and *t* is the spin diffusion time.^66,67^ For typical rigid organic solids, the ^1^H spin diffusion coefficient (*D*) is typically on the order of 10^−12^ cm^2^ s^−1^ to 10^−11^ cm^2^ s^−1^, meaning ^1^H magnetization is mathematically bounded to diffuse approximately 50 to 170 nm over 5 seconds.^66–69^ For the ^19^F saturation recovery NMR experiments, the times involved are longer (*t* ≈ 20–200 s), however, it is reasonable to expect that the ^19^F spin diffusion coefficient will be smaller than ^1^H due to greater ^19^F inter-atomic distances, the lower concentration of ^19^F, and larger ^19^F chemical shift differences. Assuming the diffusion coefficient is 1–2 orders of magnitude smaller for ^19^F than ^1^H (*D* = 10^−14^ to 10^−12^ cm^2^ s^−1^), then the domain size is approximately 10 nm to 100 nm assuming a diffusion time of 20 s. However, these are the lower bounds on the domain size, the experimental domain size could be larger. Therefore, the high-pressure polymorph likely exists as physically distinct crystallites or large segregated domains within the bulk matrix, rather than as molecular-level defects or a solid solution.^27^

This conclusion of phase segregation is further substantiated by 2D ^19^F–^19^F correlation experiments, which probe spatial proximities between fluorine nuclei. 2D ^19^F–^19^F spin diffusion NMR spectra were recorded with a short mixing time of 1 ms (Fig. 3A) and a longer 50 ms mixing time to allow for spin diffusion (Fig. 3B). The spectrum recorded with a 50 ms mixing time allows for spin diffusion to occur over longer distances. However, this spectrum only displays correlations between peaks belonging to the same phase. The absence of cross-peaks is likely not a sensitivity artifact, as the diagonal signals for the ambient phase exhibits a high signal-to-noise ratio of approximately 75. A 50 ms mixing time is sufficiently long for ^19^F magnetization to transverse several nanometers in a rigid lattice.^66,67^ Therefore, the lack of intermolecular correlations directly confirms that the fluorine atoms of the two different forms are spatially distant from one another (*i.e.*, > 1 nm).

To resolve ^1^H chemical shifts for the ambient and high-pressure phases, we performed 2D ^1^H → ^19^F CP heteronuclear correlation (HETCOR) NMR experiments (Fig. 3C and D). The standard ^1^H → ^19^F CP HETCOR experiment (Fig. 3C) relies on direct heteronuclear dipolar couplings to establish correlations. This technique selectively correlates ^19^F sites with protons in their immediate spatial proximity. Because the comprehensive chemical shift assignments for the pure ambient phase were already established in our prior work,^52,61^ the primary analytical challenge was to observe the overlapped ^1^H resonances of the minority high *P* phase and confirm their assignment. To overcome this overlap and isolate the phase-pure ^1^H NMR spectra for both components within the mixture, a 25 ms ^1^H spin diffusion period was incorporated into the pulse sequence (Fig. 3D). The 25 ms ^1^H spin diffusion period prior to the CP transfer allows magnetization to equilibrate across the proton network of each phase. This process facilitates the diffusion of ^1^H magnetization throughout the crystal lattice, correlating the ^19^F NMR signals with the bulk proton environment rather than solely the nearest neighbors.^66,68,69^ The ^19^F chemical shifts of the major and high-pressure phases are resolved, therefore, the HETCOR spectrum acts as a spectral filter, allowing for the extraction of a sub-spectrum containing only the ^1^H NMR signals associated with each specific phase. The observation of distinct ^1^H chemical shifts for each pair of ^19^F chemical shifts again suggests that the ambient phase and high *P* phase are segregated. Assuming ^1^H spin diffusion constants of 10^−11^ to 10^−12^ cm^2^ s^−1^, a 25 ms ^1^H spin diffusion delay will probe correlations over approximate length scales of 12 nm to 4 nm.

### 3.3 Measuring ^1^H chemical shifts using fast MAS double-quantum NMR experiments

**Fig. 4:**
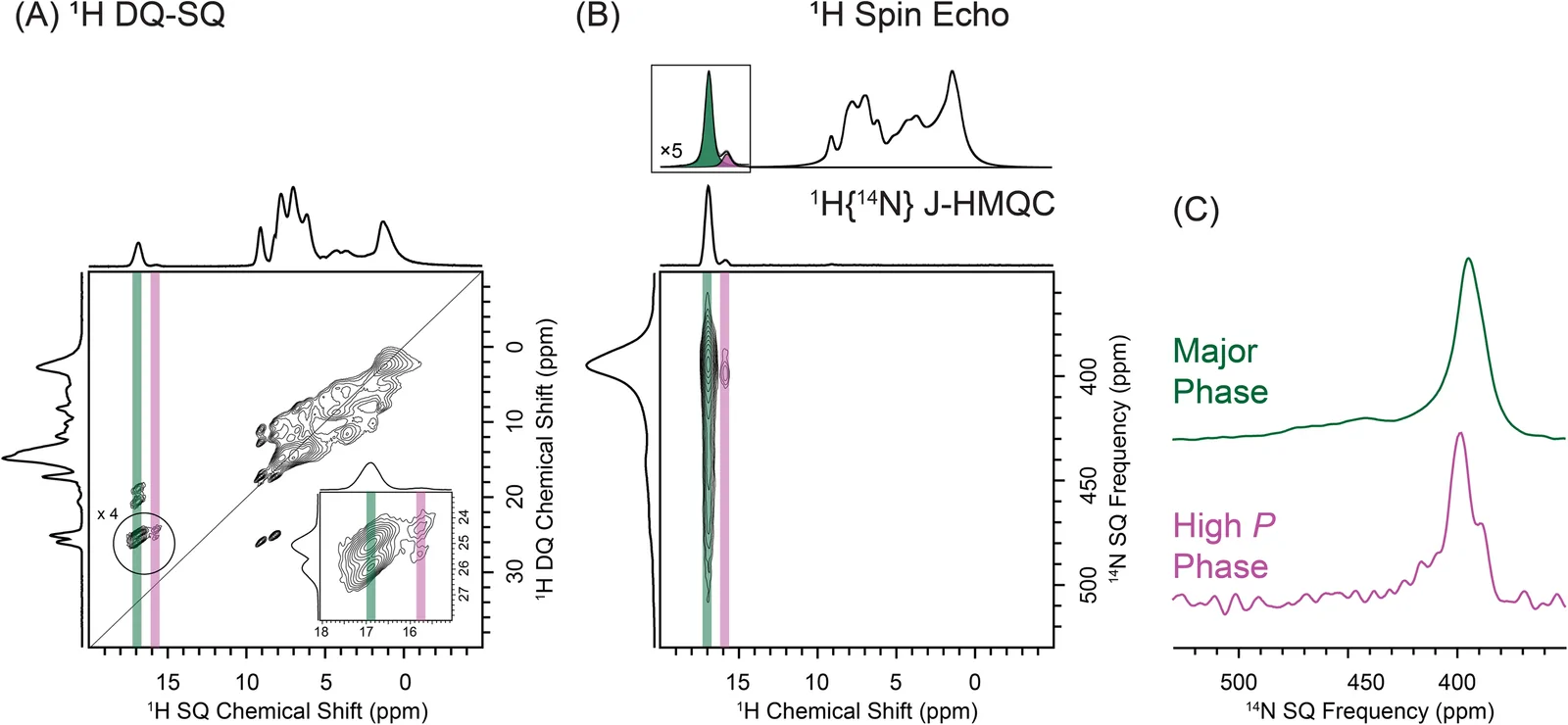
(A) 2D ^1^H DQ-SQ NMR spectrum and (B) 2D ^1^H{^14^N} *J*-HMQC SSNMR spectrum of GDC-0022 tosylate salt that was compressed with 250 MPa of pressure. A MAS frequency of 50 kHz and a magnetic field of 18.8 T was used for all experiments. (C) Comparison of extracted ^14^N SSNMR spectra of major ambient phase and high-pressure phase.

A dipole-based 2D ^1^H DQ-SQ correlation NMR spectrum of the 250 MPa compressed sample was recorded using a magnetic field of 18.8 T and a fast MAS frequecy of 50 kHz to obtain high ^1^H NMR resolution. This 2D NMR spectrum allows additional ^1^H chemical shifts to be measured and assigned for the ambient and high-pressure phases (Fig. 4A). The 2D ^1^H DQ-SQ NMR spectrum shows that the high-frequency ^1^H NMR signals from the ammonium group of each phase are correlated to two other ^1^H NMR signals with chemical shifts greater than 8.0 ppm (key correlations shown in the inset of Fig. 4A). These other ^1^H NMR signals are assigned to the CH groups of the triazole ring, based upon their high chemical shifts and because these ^1^H atoms are within a few Angstroms of the ammonium ^1^H atom. Based upon the ^1^H chemical shifts observed in the DQ dimension, the ^1^H chemical shifts of the triazole CH groups can be extracted. The ^1^H chemical shift of a CH group was determined by subtracting the ^1^H chemical shift of the ammonium NH NMR signal from the observed DQ ^1^H chemical shift (*δ*_iso_(CH) = δ_iso_(DQ) − δ_iso_(NH)). For the ambient phase this analysis gave ^1^H chemical shifts of 8.1 ppm and 9.0 ppm for the two triazole CH groups. For the high-pressure phase this analysis gives ^1^H chemical shifts of 8.6 ppm and 9.6 ppm for the triazole CH groups. As described below, these ^1^H chemical shifts are subsequently used as key constraints to identify which CSP structures match the experimental crystal structure.

### 3.4 Probing the N–H environment and protonation state

To gain deeper insight into the structural changes, particularly at the site of proton transfer between the tosylic acid and the GDC-0022 base, a ^1^H{^14^N} *J*-based heteronuclear multiple quantum coherence (HMQC) experiment was performed with a 50 kHz MAS frequency and an 18.8 T magnetic field (Fig. 4B and C). The high magnetic field and 50 kHz MAS frequency are key to improving the ^1^H and ^14^N NMR resolution. ^1^H{^14^N} HMQC NMR experiments are sensitive probes of the N–H bonding environment and can help distinguish between a salt (full proton transfer) and a cocrystal (proton sharing or hydrogen bonding).^61,70,71^ We previously measured ^1^H–^15^N and ^1^H–^14^N dipolar coupling constants for the ambient phase to confirm that it is indeed a salt.^52,61^ The 2D ^1^H{^14^N} *J*-HMQC NMR spectrum of the pressurized sample reveals two distinct correlations. A strong cross peak is observed between a ^1^H signal at 16.9 ppm and a corresponding ^14^N NMR signal, assigned to the major ambient-pressure phase. The assignment of the most intense cross-peak (^1^H at 16.9 ppm) relies on our previously published pure-phase data for the ambient-pressure GDC-0022 tosylate salt,^61^ confirming the proton is fully transferred to the nitrogen base. Conversely, the structural interpretation of the high-pressure phase is derived directly from the present ^1^H{^14^N} *J*-HMQC spectrum. The 2D ^1^H{^14^N} *J*-HMQC spectrum of the pressurized sample reveals that ^1^H NMR signal at 15.8 ppm is correlated to a ^14^N NMR signal that is similar in width and frequency to that of the ambient phase. The similarity of the ^14^N NMR signal position and lineshape implies that within the high *P* phase, the ^14^N EFG tensor parameters and chemical are nearly identical to the ambient phase. The ^14^N EFG tensor parameters and chemical shift are very sensitive to the N–H bond length,^61^ therefore we can conclude that the NH bond lengths must be nearly identical in both phases, implying that there is a protonated nitrogen atom in the high-pressure polymorph. The ^1^H chemical shift of 15.8 ppm for the high-pressure phase also falls well within the range expected for a protonated nitrogen (ammonium).

### 3.5 Structure determination by NMR crystallography

**Fig. 5:**
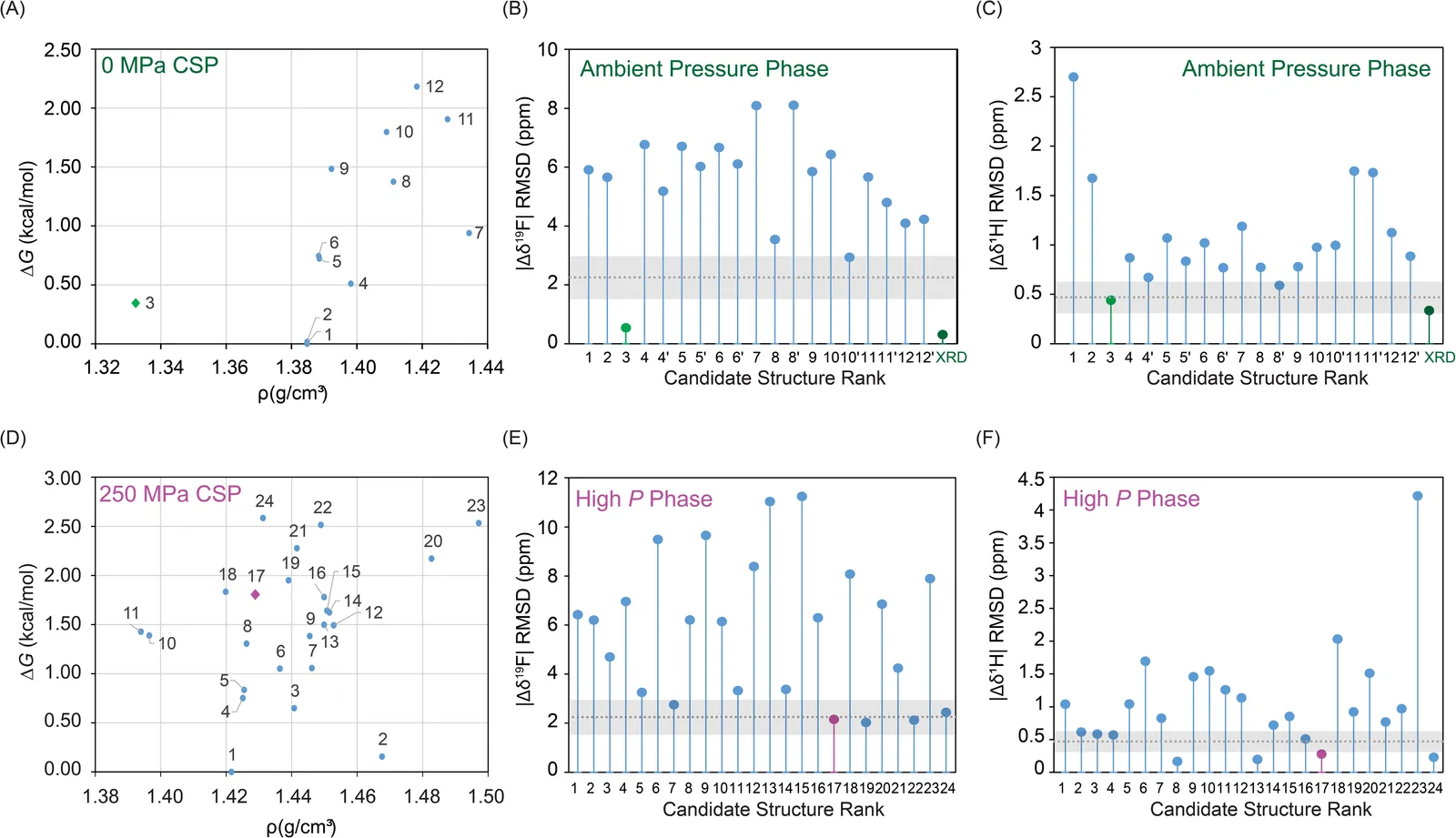
Plots of relative Gibbs-Free energy as a function of density for CSP structures output by GRACE for (A) ambient pressure (0 MPa) and (D) 250 MPa of applied pressure. Free energies are computed by GRACE and include entropic contributions from phonon calculations. Comparison of ^19^F and ^1^H RMSD of calculated chemical shifts for (B and C) the ambient pressure phase and (E and F) the high-pressure phase. The dashed horizontal lines represent the cross-validation RMSD of ^1^H calibration curve (0.47 ppm)^60^ and ^19^F calibration curve (2.23 ppm, Fig. S5 and Table S2). The grey band in (B) and (E) represent the expected 1*σ* error limits on RMSD (±0.72 ppm) derived from the calibration set. The grey band in (C) and (F) represent the standard deviation of RMSD (±0.16 ppm) for triplets of ^1^H chemical shifts derived from the previously published calibration set.^60^

With key ^1^H and ^19^F experimental chemical shifts for each phase established, CSP calculations utilizing the GRACE workflow^72^ were employed to generate candidate crystal structures for NMR crystallography. The NMR crystallography protocol was first validated using the ambient-pressure phase, which has a known single-crystal X-ray diffraction structure.^52^ The CSP calculations generated landscapes of energetically plausible crystal structures. Within the GRACE workflow structure ranking and geometry optimization are governed by the enthalpy (*H* = *U* + *PV*), explicitly accounting for the pressure-volume work term.^9^ Within GRACE phonon mode calculations are used to generate an entropy term for each structure, resulting in free energies for each structure (*G* = *H* − *TS*). Performing CSP with an applied pressure of 0 MPa yields 12 candidate structures with free energy differences of less than 2.5 kcal mol^−1^ (10.5 kJ mol^−1^, Fig. 5A and Table S3). All relative free energies reported in Fig. 5 are expressed per mole of molecules (normalized to *Z*′ = 1, corresponding to one GDC-0022 tosylate ion pair), not per mole of unit cells, ensuring that comparisons between structures with different *Z* values are made on an equivalent per-molecule basis. Fig. S4 shows the relative enthalpy and relative free energies of the 12 candidate CSP structures. If relative enthalpy is used, then the order of the structure rankings is changed, but all ambient pressure structures have a relative enthalpy within 1.6 kcal mol^−1^ of one another. The 12 candidate structures all had 1 or 2 symmetry-inequivalent molecules in their unit cells (*Z*′ = 1 or *Z*′ = 2). Crystal structures obtained from CSP were then subjected to the PBE GIPAW^56^ planewave DFT^54,73,74^ calculations, as implemented in CASTEP.^53^

The analysis here focused on the three ^1^H NMR signals (ammonium NH and two from triazole CH groups) and ^19^F NMR signals because all of these NMR signals are well resolved, definitively assigned, and exhibit high sensitivity to local structural changes (Fig. 3D and 4A). The correct structure was identified by establishing which candidate shows the best agreement between the calculated and experimental chemical shifts, as indicated by RMSD metrics.^22,38,43,45^ Standard deviations on RMSD metrics were calculated following the procedures of Zakeri and Widdifield.^75^ First, we consider the RMSD of the calculated and experimental ^19^F chemical shifts. The rank 3 structure is the only one which gives calculated ^19^F chemical shifts that are below the expected RMSD uncertainty limits of the PBE-GIPAW calculations (Fig. 5B and Table S5). One of the molecules in the rank 10 structure also gives calculated ^19^F chemical shifts which show reasonable agreement with experiment. However, the rank 10 structure has *Z*′ = 2 and the other molecule in the unit cell has a high RMSD for its calculated ^19^F chemical shifts, therefore, this candidate is disqualified. The three calculated ^1^H chemical shifts for the rank 3 structure also shows good agreement with the experiment, with RMSD below the cutoff, leading us to conclude that it is the correct structure (Table S6). Alternatively, the structures can also be evaluated by a reduced *χ*^2^ metric that combines the three calculated ^1^H chemical shifts and two calculated ^19^F chemical shifts (Table S7).^76^ Using the reduced *χ*^2^ metric indicates that the rank 3 structure has a 99.97% equal-prior posterior probability (*P*_N_) of being the correct structure (Table S7).^76^ The more conservative uniform *χ*^2^ probabililty (*P*_UC_) statistic introduced by Mueller indicates that the rank 3 structure has a 98.4% probability of being the correct structure (Table S7).^76^

**Fig. 6:**
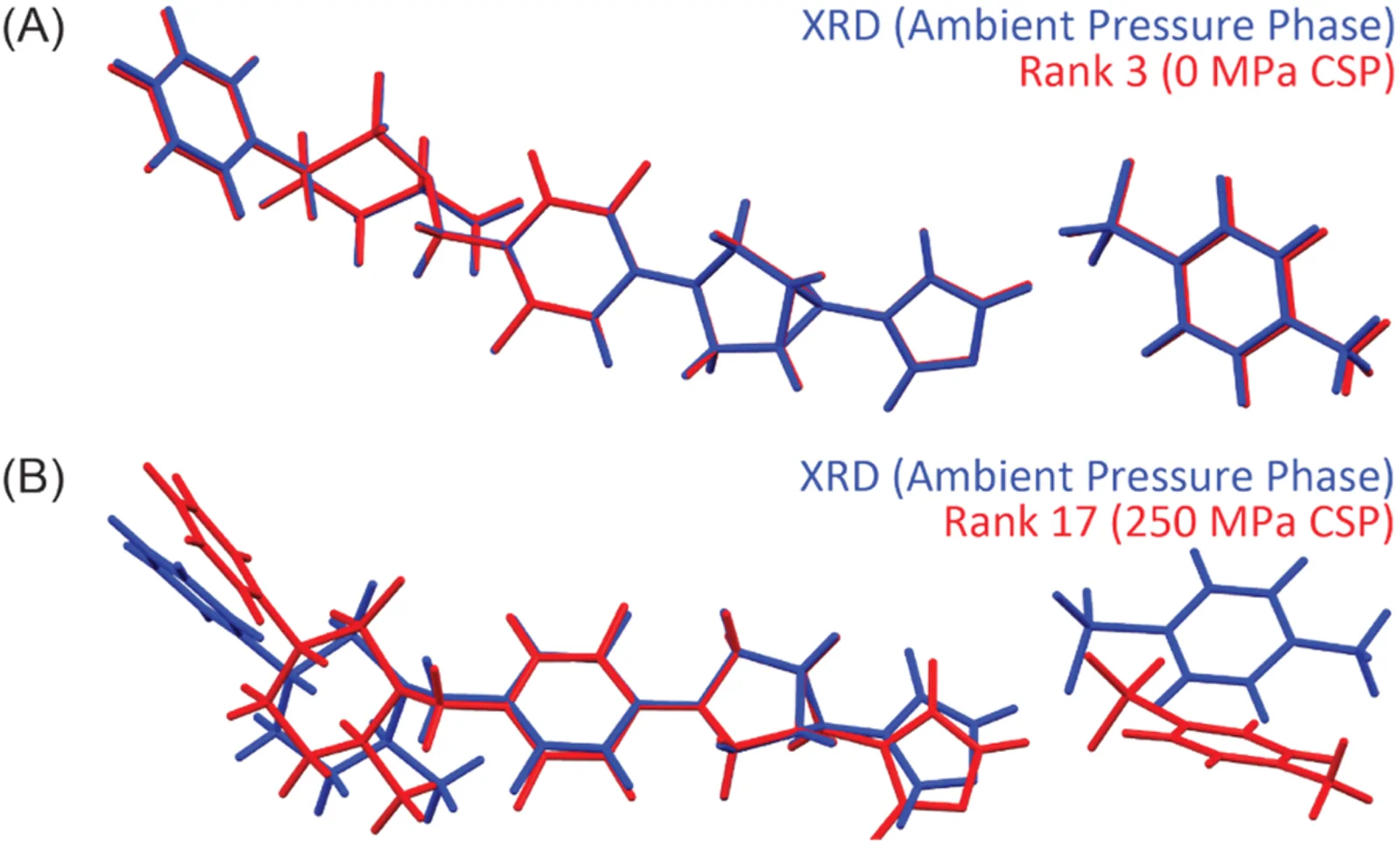
(A) Comparison of 0 MPa CSP rank 3 structure to the known single-crystal X-ray diffraction structure. (B) Comparison of 250 MPa CSP rank 17 structure to the known single-crystal X-ray diffraction structure.

A direct structural comparison of the rank 3 structure with the known single-crystal XRD structure of the GDC-0022 tosylate salt confirms that both structures are essentially identical (Fig. 6A). Consistent with the similarity of the rank 3 structure and the known single-crystal X-ray structure, the calculated ^19^F chemical shifts and ^1^H chemical shifts for both structures are nearly identical, with both structures also giving similar RMSD values (Fig. 5B, C, Tables S5 and S6). The successful identification of the known crystal structure of the ambient phase demonstrates the accuracy and reliability of the integrated CSP-NMR methodology, even though we use only two predicted ^19^F chemical shifts and three ^1^H chemical shifts to identify the correct candidate structure.

The CSP energy landscape provides a clear thermodynamic rationale for the observed pressure-induced phase transformation. As shown in Fig. 5A, the rank 3 structure is the least dense of all predicted structures with a density of 1.33 g cm^−3^, explaining its instability under compression. All other predicted structures have densities greater than 1.39 g cm^−3^, with the densest structure (rank 7) having a density greater than 1.43 g cm^−3^. The molar volumes of all other structures are reduced by at least 19.1 cm^−3^ mol^−1^ as compared to the rank 3 structure (*V*_m_ = *M*/ρ, where *ρ* is density, and *M* is the molar mass, 671.79 g mol^−1^). An applied pressure of 250 MPa will increase the free energy/enthalpy of the rank 3 structure by 1.3 kcal mol^−1^ (5.5 kJ mol^−1^) relative to a phase with a higher density of 1.39 g cm^−3^. Therefore, it is plausible that with an applied pressure of 250 MPa the Gibbs free energy of the rank 3 structure could become more positive relative to a denser phase, making a phase transition exergonic.

To determine the structure of the high-pressure phase, an independent CSP run was performed with 250 MPa of applied pressure, rather than applying a post-hoc *PV* correction to the 0 MPa structures. Within the GRACE workflow, structure generation and plane-wave DFT geometry relaxation are performed against the full enthalpy *H* = *U* + *PV* at the target pressure,^9,72^ ensuring that pressure-driven changes in molecular conformation and crystal packing are explicitly captured. A post-hoc *PV* enthalpy correction applied to 0 MPa-relaxed structures would not account for these structural reorganizations, as high-pressure geometry optimization is known to yield structurally distinct candidates from ambient-pressure relaxations.^77,78^ Consequently, CSP with a pressure of 250 MPa was used determine the structure of the high-pressure phase. For the high-pressure CSP *Z*′ was restricted to 1 due to computational constraints. CSP at a pressure of 250 MPa results in 24 candidate structures with relative free energy differences of less than 2.6 kcal mol^−1^ (Fig. 5D and Table S4). Ranking of the structures by relative free energy and relative enthalpy is shown in Fig. S4. Notably, with the higher applied pressure of 250 MPa during the CSP step, there is an increase in the density of the candidate structures, with the least dense structure having a density of 1.39 g cm^−3^ and the densest structure having a density over 1.49 g cm^−3^. The two ^19^F chemical shifts and three ^1^H chemical shift were then calculated for the 24 candidate structures and compared to experimental results (Fig. 5E, F, Tables S8 and S9).

Of these 24 candidate structures, the rank 7, 17, 19, 22 and 24 structures have calculated ^19^F chemical shifts that are near or below the RMSD cutoff. However, out of these 5 candidates, only structures 17 and 24 gave calculated ^1^H chemical shifts with RMSD below the cutoff. A structural overlay of the rank 17 and rank 24 candidate structures reveals an extremely high degree of molecular conformational similarity (Fig. S8), which accounts for their virtually identical calculated NMR parameters.^79^ Structure 24 (*P*2_1_2_1_2_1_, *Z* = 4, *V* ≈ 3118 Å^3^) has a unit cell volume approximately twice that of structure 17 (*P*2_1_, *Z* = 2, *V* ≈ 1562 Å^3^), with the number of molecules per unit cell doubled in structure 24. Although the two structures differ in space group symmetry, *P*2_1_ is a subgroup of index 2 of *P*2_1_2_1_2_1_. Structure 24 therefore does not represent a genuinely distinct polymorph^15^ from structure 17 for the purposes of NMR-based structure discrimination. Additionally, structure 17 retains the (*P*2_1_) space group symmetry of the ambient phase, suggesting a plausible isosymmetric transition driven by density increase rather than a reconstructive symmetry change.^10,80^ Consequently, while the high-pressure search was constrained to *Z*′ = 1 for computational feasibility,^15–17,81^ the excellent agreement between calculated and experimental NMR parameters strongly suggests that the rank 17 structure is the best representation of the high-pressure phase. Analyzing the *χ*^2^ ranking of all high-pressure CSP structures gives a *P*_N_ of 96.1% that the rank 17 structure is the correct structure (Table S10). If the more conservative *P*_UC_ is used,^76^ then the rank 17 structure has an 88.8% probability of being the correct structure. The rank 14 structure is the next most probable, but it has *P*_UC_ of only 2.07%. Therefore, we conclude that the rank 17 structure is assigned as the definitive high-pressure structure.

The experimental conversion of the ambient phase to high-pressure phase is observed to be incomplete with applied pressures of 250 MPa to 1800 MPa (conversion of 14–23% of the ambient phase). This partial transformation could arise from the kinetically hindered nature of the solid–solid phase transition and the likely inhomogeneous stress distribution within the powder compact.^7^ The persistence of the high-pressure phase upon decompression further indicates that it is kinetically trapped, prevented from relaxing back to the ambient form by the significant activation barrier required for cooperative molecular reorientation.^6,82^ Furthermore, comparison of the lattice parameters for the known ambient-phase structure and the rank 17 CSP structure indicates that this densification is primarily attributable to a significant compression along the crystallographic *c* axis, which decreases from approximately 36.53 Å in the ambient phase to 32.84 Å in the high-pressure form. This anisotropic behavior is consistent with the mechanism of void reduction often observed in high-pressure molecular crystallography,^12,83^ where the structure compresses preferentially along directions dominated by weaker interactions and interstitial voids.

A complementary strategy to probe the structure of the high-pressure phase would involve performing *in situ* high-pressure powder X-ray diffraction (PXRD) experiments.^84^ Collecting a diffraction pattern at pressures sufficient to induce the transformation would provide an experimental benchmark for the high-pressure phase. Alternatively, to overcome the potential lack of large single crystals for the high-pressure form, Three-Dimensional Electron Diffraction (3D-ED) could be applied to solve the crystal structure directly from microcrystalline particles.^4,85–87^ Subsequent Rietveld refinement using the 3D-ED derived structure^88^ or structure 17 as a comparative model would confirm the correct polymorph based on the superior quality of fit to the experimental data.^89^ Furthermore, because the high-pressure form was characterized as a minority component within a mixture, its bulk physicochemical properties in a pure state, such as solubility, dissolution rate, and stability at ambient pressure remain unknown. Future research should therefore focus on methods to isolate the pure high-pressure phase. Once a pure sample is obtained, its dissolution profile can be systematically measured to assess any potential impact on bioavailability.^90^ Additionally, variable-temperature and variable-pressure experiments, such as high-pressure differential scanning calorimetry (DSC), could be used to construct a pressure–temperature (*P*–*T*) phase diagram.^91^ Such a diagram would definitively establish the thermodynamic relationship (monotropic or enantiotropic) between the major and high-pressure forms,^92^ providing critical information for controlling the solid form during manufacturing.

## 4 Conclusions

This work has successfully characterized a pressure-induced polymorphic transformation of the GDC-0022 tosylate salt, identifying a new high-pressure phase that forms when a powdered sample is subjected to pressures above 50 MPa. Interestingly, approximately 14% to 23% of the sample remains in the presumably metastable high-pressure phase after the powder is brought back to ambient pressure. ^19^F NMR experiments performed over an 18 months time span suggest that the high-pressure phase remains stable under ambient temperature and humidity. An integrated NMR crystallography workflow proved to be a powerful tool for analyzing this complex, heterogeneous system. NMR experiments confirmed that both polymorphs are salts and that the pressure induced transformation results in physically segregated crystalline domains. By leveraging two-dimensional correlation SSNMR experiments, the distinct spectroscopic signatures of the ambient phase and high-pressure phases were separated. These experimental constraints were then used in conjunction with CSP and DFT calculations to validate the known structure of the ambient-pressure phase and to identify a high-density, structurally similar candidate for the previously uncharacterized high-pressure polymorph. CSP at ambient pressure suggests the low relative density of the known ambient phase crystal structure of the GDC-0022 tosylate salt explains why it undergoes a pressure driven phase transition.

More generally, our results highlight the utility of ^19^F chemical shifts for NMR crystallography. The study highlights a robust, generally applicable framework for the structural elucidation of minority phases within complex solid mixtures. This capability could be of use to the pharmaceutical industry for understanding and mitigating risks associated with unintended phase transitions during drug manufacturing. Furthermore, this work establishes that pressurization could be a useful tool to explore the free energy landscape of solid drug forms.

## Conflicts of interest

There are no conflicts to declare.

## Supplementary Material

SC-017-D6SC02621D-s001

## Data Availability

The experimental raw NMR data and computational result are openly available in a Zenodo archive at https://doi.org/10.5281/zenodo.21462920. Supplementary information (SI): PXRD patterns, pulse sequence diagrams, additional NMR spectra, tables of calculated chemical shifts, and statistical ranking of CSP structures. See DOI: https://doi.org/10.1039/d6sc02621d.
