# Supplementary material for: NMR crystallography of the high-pressure form of a multi-component active pharmaceutical ingredient
Source: Chem Sci. 2026 Jul 29. Online ahead of print. doi: 10.1039/d6sc02621d (PMC13417174; doi:10.1039/d6sc02621d)
Supplement: SC-OLF-D6SC02621D-s001 [file SC-OLF-D6SC02621D-s001.pdf]

Supplementary Information (SI) for Chemical Science.
This journal is © The Royal Society of Chemistry 2026

***Supplementary Information for:***

# NMR Crystallography of the High-Pressure Form of a Multi-Component Active Pharmaceutical Ingredient

Jiashan Mi,[1] Amrit Venkatesh,[2,3] Ivan Hung,[2] Nathan Ho,[4] Antonio G. DiPasquale,[4*] Joseph W. Lubach,[4*] Aaron J. Rossini,[1*]

[1]Department of Chemistry, Iowa State University, Ames, IA 50010, United States

[2]National High Magnetic Field Laboratory, Florida State University, Tallahassee, FL 32310, United States

[3]Department of Chemistry, University of Virginia, Charlottesville, VA 22904, United States

[4]Genentech, Inc., Department of Small Molecule Pharmaceutical Sciences, 1 DNA Way, South San Francisco, CA 94080, United States

AUTHOR INFORMATION

**Corresponding Author**

*e-mail: arossini@iastate.edu, phone: +1 515-294-8952.
*e-mail: josephwl@gene.com, phone: +1 650-225-5072.
*e-mail: dipasqua@gene.com, phone: +1 650-491-7170.

**Table of Contents**


| **Supplementary Texts** | |
|---|---|
| Methods: Detailed Solid-State NMR Acquisition Parameters | S3-5 |
| | |
| **Supplementary Figures** | |
| Figure S1. Comparison of experimental and calculated PXRD patterns. | S6 |
| Figure S2. NMR pulse sequences. | S7 |
| Figure S3. Comparison of $^{13}C$ CPMAS SSNMR spectra of the ambient-pressure phase and the sample compressed at 250 MPa. | S8 |
| Figure S4. Comparison of relative Gibbs free energy and relative enthalpy for CSP structures generated with applied pressures of 0 MPa and 250 MPa. | S9 |
| Figure S5. $^{19}F$ chemical shift calibration plot. | S10 |
| Figure S6. $^{19}F$ SSNMR spectra of GDC-0022 tosylate salt compressed at 1800 MPa. | S11 |
| Figure S7. Comparison of $^{19}F$ SSNMR spectra of the 250 MPa sample acquired 18 months apart. | S12 |
| Figure S8. Structural overlay of CSP Rank 17 and Rank 24 calculated at 250 MPa. | S13 |
| Figure S9. $^{19}F$ SSNMR spectra of GDC-0022–phosphoric acid cocrystal. | S13 |
| | |
| **Supplementary Tables** | |
| Table S1. GIPAW Calculated $^{19}F$ Chemical Shifts | S14 |
| Table S2. GIPAW Calculated $^{1}H$ Chemical Shifts of Theophylline and Histidine. | S15 |
| Table S3. Crystallographic parameters and relative free energies of the 12 calculated structures at 0 MPa. | S16 |
| Table S4. Crystallographic parameters and relative free energies of the 24 calculated structures at 250 MPa. | S17-18 |
| Table S5. DFT-predicted $^{19}F$ solid-state NMR isotropic magnetic shieldings and chemical shifts for the 12 candidat structures generated by CSP at 0 MPa. | S19 |
| Table S6. DFT-predicted $^{1}H$ solid-state NMR isotropic magnetic shieldings and chemical shifts for the 12 candidate structures generated by CSP at 0 MPa. | S20 |
| Table S7. Statistical evaluation of the agreement between experimental and DFT-calculated solid-state NMR chemical shifts for the 12 candidate structures at 0 MPa. | S21 |
| Table S8. DFT-predicted $^{19}F$ solid-state NMR isotropic magnetic shieldings and chemical shifts for the 24 candidate structures generated by CSP at 250 MPa. | S22 |
| Table S9. DFT-predicted $^{1}H$ solid-state NMR isotropic magnetic shieldings and chemical shifts for the 24 candidate structures generated by CSP at 250 MPa. | S23 |
| Table S10. Statistical evaluation of the agreement between experimental and DFT-calculated solid-state NMR chemical shifts for the 23 high-presssure candidate structures. | S24 |

***Solid-State NMR Spectroscopy Experiments.*** SSNMR experiments were conducted using four different spectrometers with magnetic field strengths of 9.4 T (Iowa State University), 11.7 T (Genentech, Inc.), and 14.1 T and 18.8 T (National High Magnetic Field Laboratory (NHMFL)). All experiments were conducted at ambient temperature and pressures, without any active cooling of the rotor to compensate for frictional heating of rotors during MAS. Chemical shifts were referenced to neat tetramethylsilane (TMS) via a secondary solid adamantane standard ($\delta$ $^1$H = 1.82 ppm) for $^1$H.[1] $^{19}$F chemical shifts were referenced indirectly to trichlorofluoromethane ($CFCl_3$) ($\delta_{iso}$ = 0.0 ppm) using the IUPAC relative frequency ratio.[1] All $^{14}$N spectra were referenced to the nitromethane scale ($CH_3NO_2$ $\delta$ = 0.0 ppm). All SSNMR spectra were processed using the TopSpin v3.6.4 software package. A schematic illustration of all pulse sequences is given in Figure S2.

*9.4 T Experiments (ISU):* The 1D $^{19}$F spin echo experiment (Figure 1D) was performed on a Bruker Avance III HD spectrometer operating at a magnetic field strength of 9.4 T ($\nu_0$ ($^{19}$F) = 376.6 MHz). The experiment was conducted using a 1.3 mm HX MAS probe configured for $^{19}$F operation. The sample was packed into a 1.3 mm zirconia rotor and spun at a MAS frequency of 50 kHz. The spectrum was acquired using a rotor-synchronized spin echo pulse sequence with a recycle delay of 30.0 s and 128 scans. The $^{19}$F $\pi/2$ pulse length was 4.0 μs, corresponding to a radiofrequency (rf) field strength of ca. 62.5 kHz.

*11.7 T Experiments (Genentech, Inc.):* $^1$H→$^{19}$F cross-polarization magic-angle spinning (CPMAS) experiments (Figure 1B) were performed on a Bruker Avance III HD spectrometer operating at a magnetic field strength of 11.7 T ($\nu_0$ ($^1$H) = 500.1 MHz). Experiments were conducted using a 4 mm HFC MAS probe (Bruker) operating in double-resonance mode. Samples were packed into 4 mm zirconia rotors and spun at a MAS frequency of 14 kHz. The CPMAS experiments were acquired with 512 scans and a recycle delay of 2.0 s. The $^1$H $\pi/2$ pulse length was 3.35 μs, corresponding to an rf field strength of ca. 75 kHz. A contact time of 1.0 ms was used. During the cross-polarization step, the $^1$H spin-lock pulse was linearly ramped from 50% to 100% amplitude to maintain the Hartmann–Hahn match condition. SWf-TPPM heteronuclear

decoupling[2] with a rf field strength of ca. 75 kHz was applied on the $^{1}H$ channel during $^{19}F$ NMR signal acquisition.

*14.1 T Experiments (NHMFL):* Relaxation and 2D correlation experiments (Figures 2 and 3) were performed on a $^{1}H$ Bruker Avance III spectrometer and a 4 mm magic angle spinning (MAS) HFX probe using a diplexer and $^{1}H/^{19}F$ band-pass filters ($\nu_0$ ($^{1}H$) = 600.6 MHz, $\nu_0$ ($^{19}F$) = 564.6 MHz). Samples were packed into Bruker 4 mm zirconia rotors with Vespel caps and spun at 10 kHz MAS frequency. Pulses ($\pi/2$ and $\pi$) on $^{1}H$ and $^{19}F$ channels used ca. 48 kHz and 53 kHz rf powers, respectively. $^{1}H\rightarrow^{19}F$ CP was performed with a spin-lock contact time of 2.0 ms, where the $^{1}H$ spin-lock pulse was linearly ramped from 80 to 100% amplitude to broaden the Hartman−Hahn match condition.[3-4] The $^{1}H$ and $^{19}F$ rf fields during spin-lock were ca. 55 kHz and 47 kHz, respectively. $^{1}H$ and $^{19}F$ spin-lattice relaxation ($T_1$) decay curves (Figure 2) were measured using saturation recovery sequences and a recycle delay of 0.5 s. The $^{1}H\rightarrow^{19}F$ CP saturation recovery experiments used 8 scans per increment, while direct $^{19}F$ saturation recovery experiments used 2 scans. 2D $^{19}F$–$^{19}F$ spin diffusion spectra (Figure 3A, B) were recorded with 8 scans per $t_1$ increment, 48 points in the indirect dimension, a rotor-synchronized indirect dimension spectral width of 10 kHz (D$t_1$ = 100.0 ms) and a recycle delay of 7.0 s. In these spin diffusion experiments, the $^{1}H\rightarrow^{19}F$ CP step was followed by $^{19}F$ mixing periods of 1 ms or 50 ms. 2D $^{1}H\rightarrow^{19}F$ CP HETCOR spectra (Figure 3C) were acquired with 16 scans per increment, 160 points in the indirect dimension, an indirect dimension spectral width of 31.25 kHz (corresponding to D$t_1$ = 32.0 ms, the duration of a single *e*DUMBO$_{1\text{-}22}$ pulse), a CP contact time of 0.6 ms, and a recycle delay of 7.0 s. For the spin-diffusion $^{1}H\rightarrow^{19}F$ CP HETCOR experiment (Figure 3D), a 25 ms $^{1}H$ spin diffusion period was inserted prior to the CP step to enable all $^{1}H$ signals of the two phases to be observed. This experiment was also acquired with 16 scans and 160 points in the indirect dimension. *e*DUMBO$_{1\text{-}22}$ homonuclear decoupling[5] was applied during the $t_1$-evolution period to enhance the resolution of the $^{1}H$ NMR spectrum. Each eDUMBO$_{1-22}$ pulse was 32 μs in duration and used a 70 kHz $^{1}H$ RF field. SPINAL-64 heteronuclear decoupling[6] was applied at ca. 50 kHz rf on the $^{1}H$ channel during signal acquisition for all $^{19}F$-detected experiments. The $^{1}H$ preamplifier was bypassed during $^{19}F$-detected experiments to improve rf efficiency.

*18.8 T Experiments (NHMFL):* High-field experiments were performed on a Bruker Avance NEO spectrometer operating at a magnetic field strength of 18.8 T ($\nu_0$ ($^{1}H$) = 799.8 MHz, $\nu_0$ ($^{14}N$) = 57.8 MHz). All experiments were conducted using a 1.3 mm HXY MAS probe (designed

and constructed at the NHMFL) configured for $^{1}H$-$^{14}N$ double-resonance operation. Samples were packed into 1.3 mm zirconia rotors and spun at a MAS frequency of 50 kHz. The 1D $^{1}H$ spin echo spectrum (Figure 1C) was acquired using a rotor-synchronized spin echo sequence with a recycle delay of 18.0 s to ensure full relaxation. The experiment utilized $^{1}H$ pulses with an RF field strength of ca. 83 kHz. The homonuclear dipolar $^{1}H$ double quantum single quantum (DQ-SQ) correlation spectra (Figure 4A) was acquired with a 50 kHz MAS frequency. $^{1}H$ $\pi/2$ pulses were 3.0 μs in duration, corresponding to an 83 kHz RF field. The rotor-synchronized Back to Back (BABA) pulse sequence was used to acquire the 2D NMR spectrum.[7] The DQ excitation and reconversion periods were each two rotor periods in duration, corresponding to 40.0 μs. The 2D NMR spectrum was acquired with 16 scans per $t_1$ increment for a total of 256 increments in the indirect dimension, utilizing a recycle delay of 4.0 s. The indirect dimension spectral width was 50 kHz, corresponding to $\Delta t_1$ = 20 μs. The 2D $^{1}H\{^{14}N\}$ *J*-HMQC experiment (Figure 4B and 4C) was acquired with a frequency-selective *J*-HMQC pulse sequence to enhance sensitivity.[8] Prior to the application of the DANTE pulse train, a broadband composite π-pulse ($\pi/2_Y$-$\pi_X$- $\pi/2_Y$) was applied to invert all $^{1}H$ magnetization. Initial transverse $^{1}H$ magnetization was excited with a rotor-synchronized DANTE pulse train consisting of seven 0.4 μs pulses that were separated by 19.6 μs. The $^{1}H$ transmitter offset was set so that the DANTE pulse train offset sat in between the $^{1}H$ NMR signal of the ammonium groups from the ambient phase and high-pressure phase. The central $^{1}H$ π pulse was 6 μs in duration corresponding to an 83 kHz RF field strength. Rotor-synchronized 20.0 μs duration excitation pulses were applied on the $^{14}N$ channel. The power of these pulses was experimentally optimized to yield maximum signal. The $^{14}N$ RF field for these pulses was not calibrated, but the $^{14}N$ pulse RF field was estimated to be around 30 kHz for these pulses. The total duration of the central spin echo in the *J*-HMQC pulse sequence was 2.04 ms (102 rotor periods). The 2D NMR spectrum was acquired with 256 scans per $t_1$ increment, a recycle delay of 0.8 s, and 128 points in the indirect $^{14}N$ dimension. The duration of the central spin echo was incremented as the $^{14}N$ $t_1$-evolution time was incremented. The indirect dimension spectral width was 25 kHz, corresponding to $\Delta t_1$ = 40 μs.

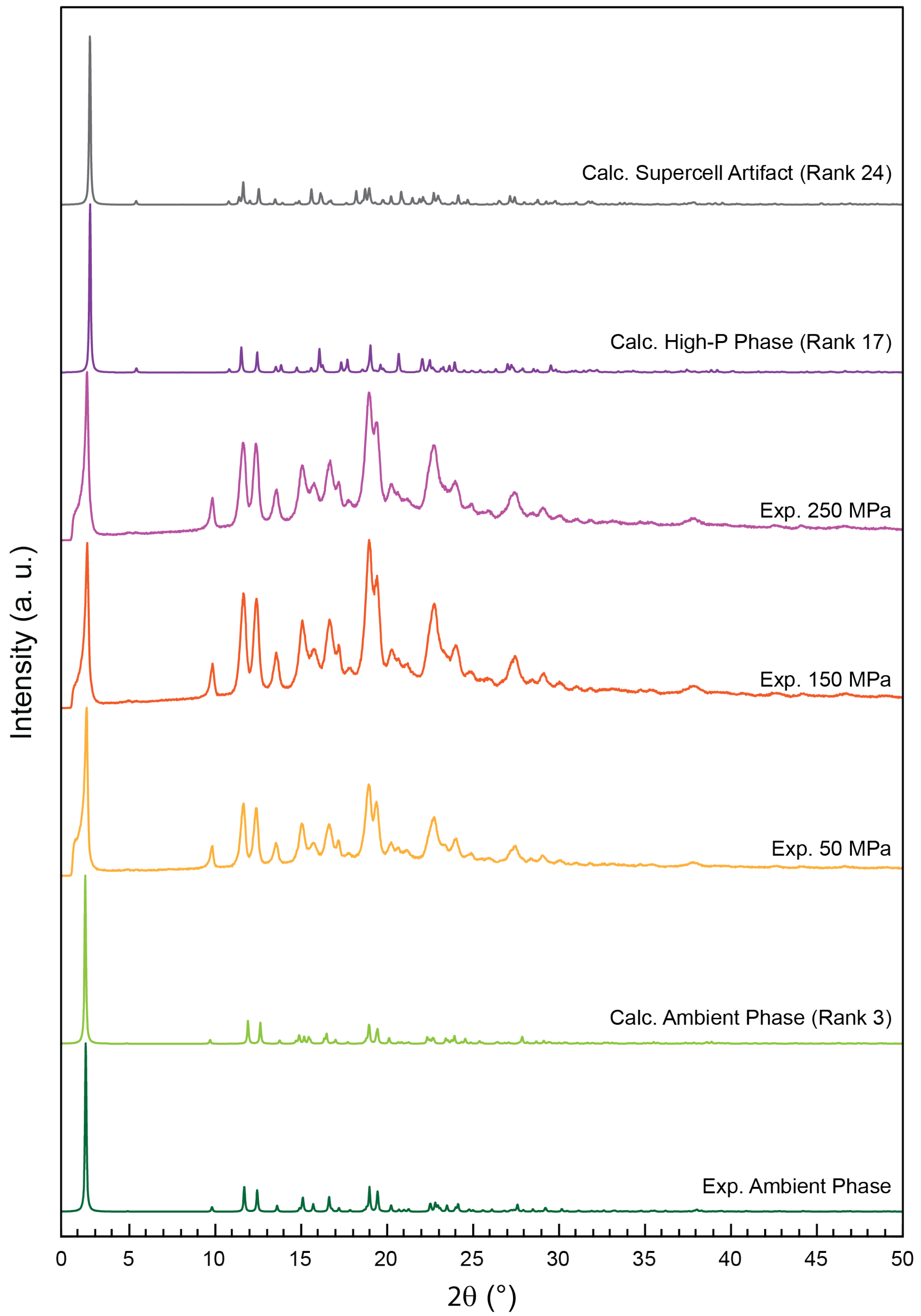


**Figure S1**. Comparison of experimental and calculated PXRD patterns. The ambient phase is shown in green (dark green: experimental; light green: calculated Rank 3). The experimental compressed samples (50–250 MPa) are shown in orange and magenta, illustrating the pressure-induced structural evolution. The calculated high-pressure candidates are depicted in purple (Rank 17) and gray (Rank 24). The experimental patterns for compressed samples exhibit peak broadening and a low-angle shoulder (~2°), attributed to lattice strain and disorder induced by mechanical processing. Due to the limited resolution and significant peak overlap in the powder diffraction data, solid-state NMR spectroscopy was utilized for definitive phase identification and structural assignment.

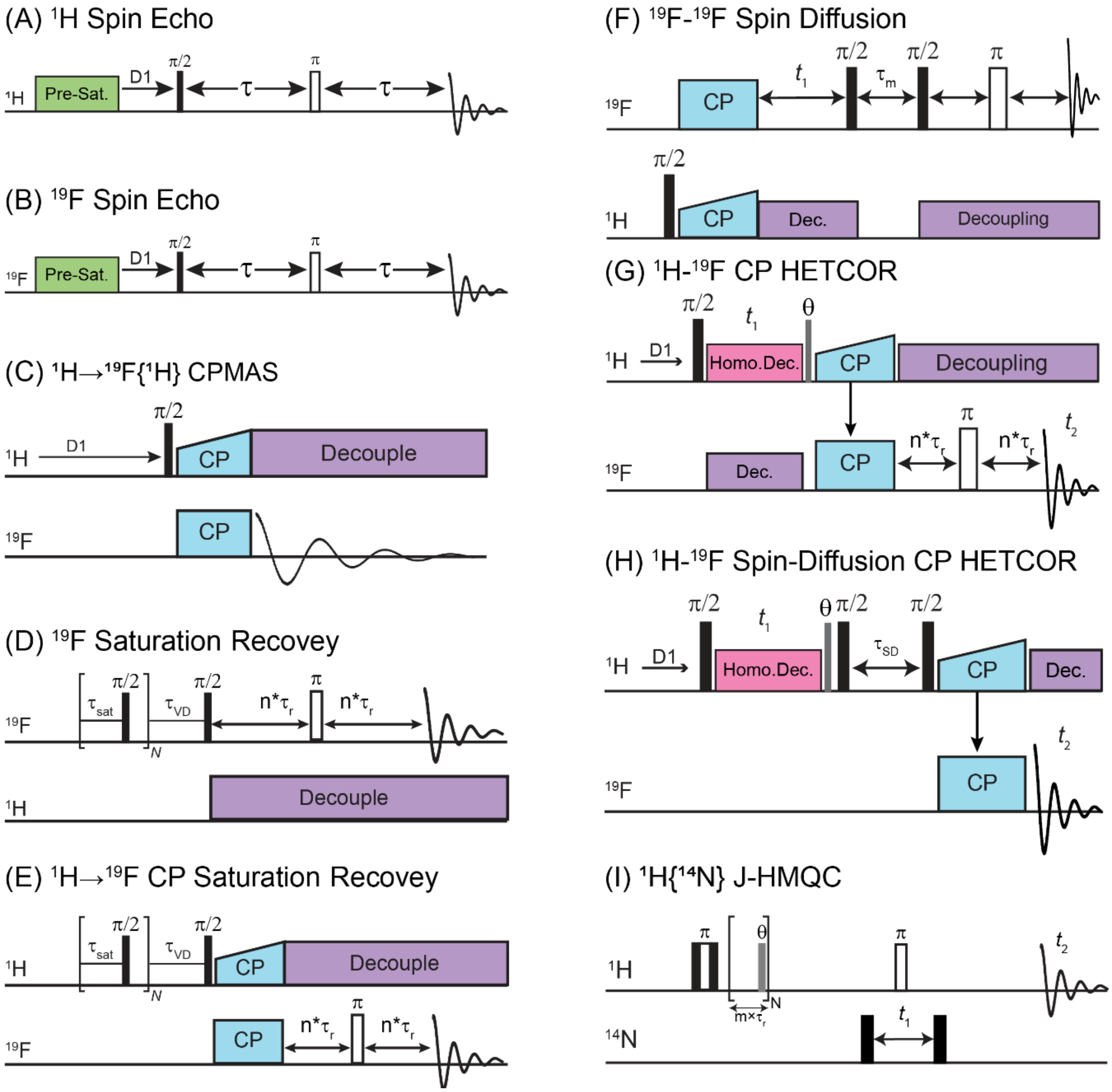


**Figure S2.** Diagrams of the pulse sequences used in this work. (A) $^{1}$H spin echo, (B) $^{19}$F spin echo, (C) $^{1}$H–$^{19}$F{$^{1}$H} CPMAS, (D) $^{19}$F saturation recovery, (E) $^{1}$H–$^{19}$F CP saturation recovery, (F) $^{19}$F–$^{19}$F CP spin-diffusion, (G) $^{1}$H–$^{19}$F CP HETCOR, (H) $^{1}$H–$^{19}$F CP spin-diffusion HETCOR, (I) Frequency-selective $^{1}$H{$^{14}$N} *J*-HMQC.

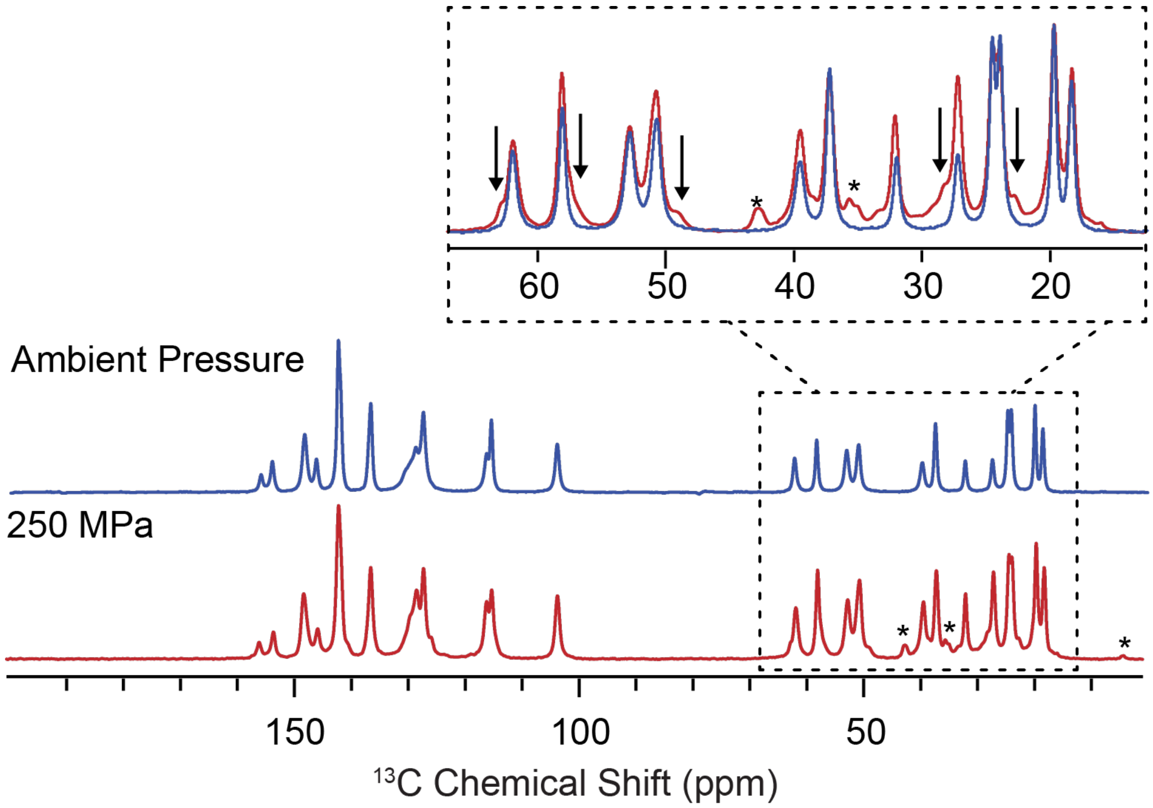


**Figure S3.** Comparison of room temperature $^{13}$C CPMAS SSNMR spectra of the ambient phase (top) and a sample that was compressed at 250 MPa (bottom). The ambient phase NMR spectrum is adapted from Figure 1F of our previous work (Zhao et al., Cryst. Growth Des., 2018). The spectrum of the ambient sample was acquired with sideband suppression (TOSS), while sidebands in the 250 MPa spectrum are marked with asterisks. The inset highlights peaks in the aliphatic region, with additional peaks in the pressurized sample indicated with arrows. The overlap of $^{13}$C NMR signals precludes unambiguous resonance assignment for the minor high-pressure phase.

(A)

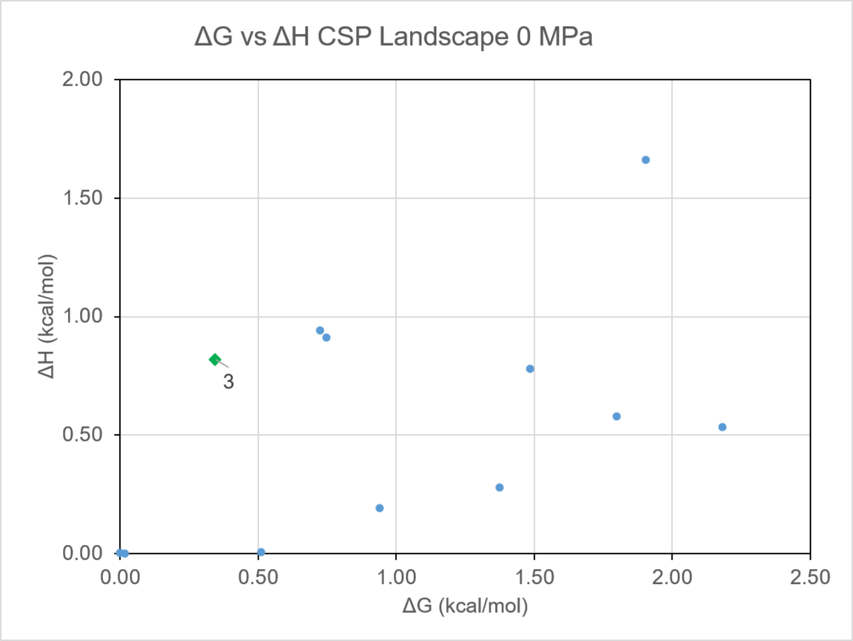


(B)

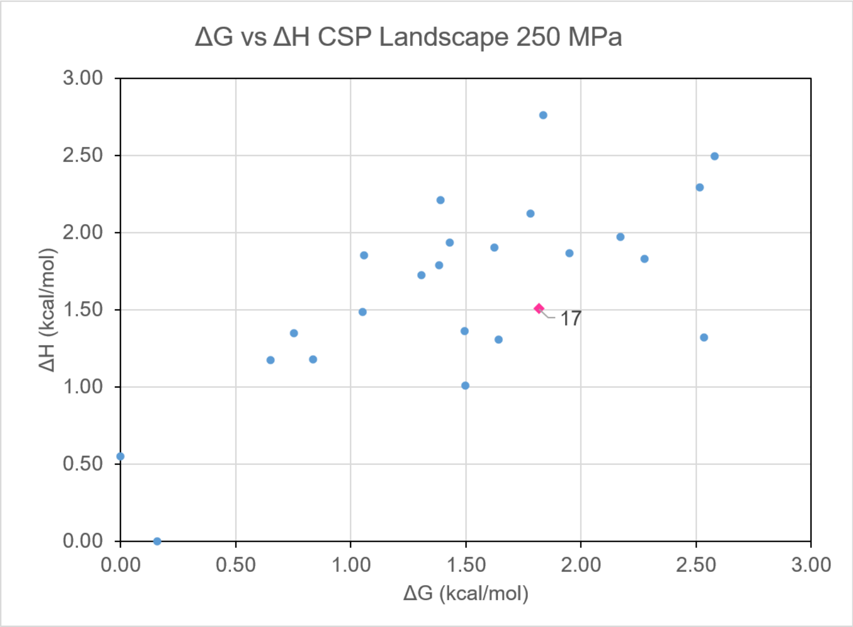


**Figure S4**. Comparison of relative Gibbs free energy ($\Delta G$) and relative enthalpy ($\Delta H$) for CSP structures generated with applied pressures of (A) 0 MPa and (B) 250 MPa. The rank 3 structure is highlighted on the 0 MPa CSP landscape, while the rank 17 structure is highlighted on the 250 MPa CSP landscape.

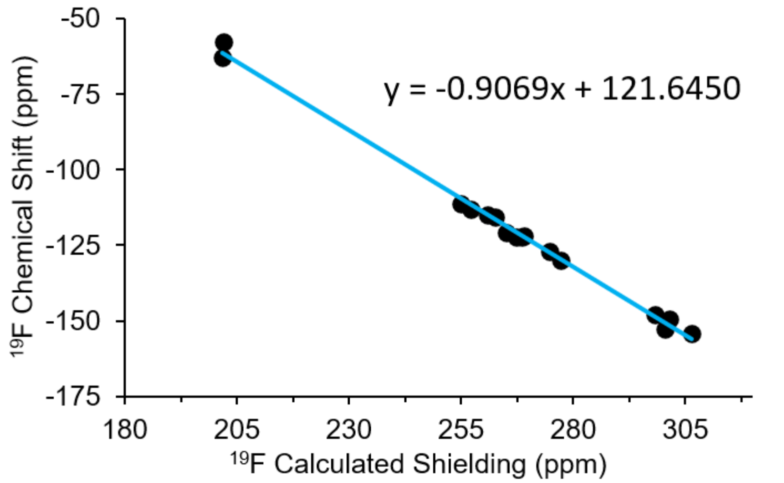


**Figure S5.** Calibration plot used to convert calculated isotropic $^{19}F$ magnetic shielding values to isotropic $^{19}F$ chemical shifts. Exact shift values and the compounds used are given in Table S1. All compounds had previously reported crystal structures that were geometry optimized before the NMR calculations to more accurately locate the hydrogen positions. The blue linear fit represents the mean calibration curve $\delta_{iso} = (-0.9069 \pm 0.0086)\sigma_{iso} + (121.6450 \pm 2.3799)$ ppm obtained via leave-one-out cross-validation (LOOCV), which yields a cross-validation RMSD of 2.23 ppm, consistent with previously reported benchmarks for periodic GIPAW DFT-based $^{19}F$ chemical shift predictions.[9-10]

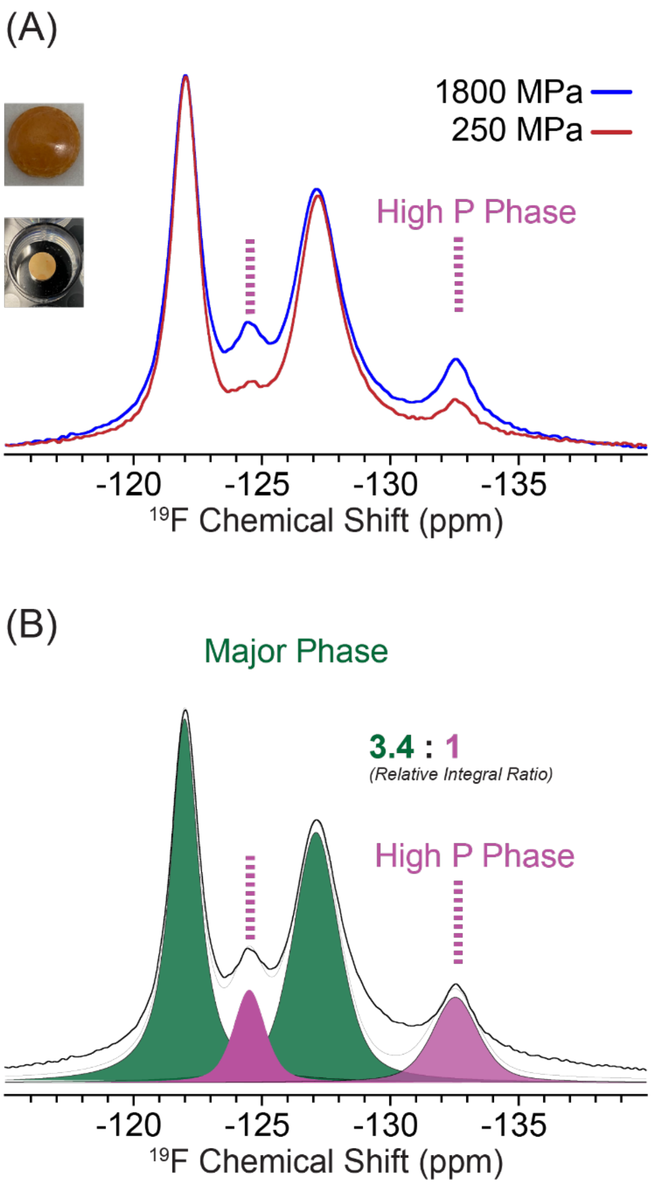


**Figure S6.** (A) $^{19}F$ spin echo NMR spectra of GDC-0022 tosylate salt compressed at 1800 MPa (blue trace) and 250 MPa (red trace). The sample compressed at 1800 MPa was held at pressure for 30 minutes using a Carver press. The insets show optical images of the compressed pellets. (B) Deconvolution of the $^{19}F$ spin echo NMR spectrum of the sample compressed at 1800 MPa. The relative integral ratio of the ambient pressure phase (Major Phase, green) to the high-pressure polymorph (High P Phase, purple) is 3.4:1, indicating that approximately 23% of the sample has converted to the high-pressure phase. All $^{19}F$ NMR spectra were acquired with a 50 kHz MAS frequency and a magnetic field of 9.4 T. Recycle delays of 30 seconds were used to acquire both NMR spectra.

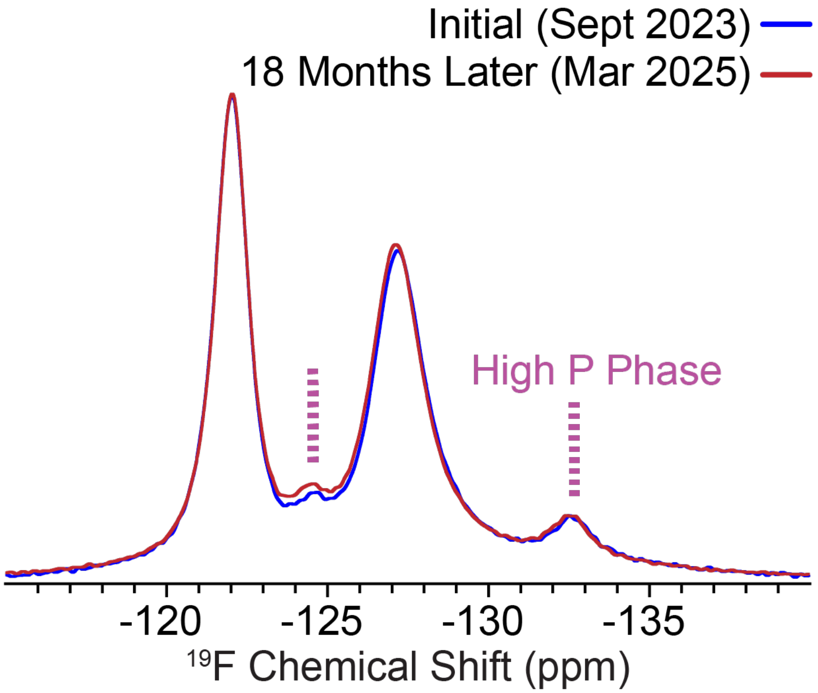


**Figure S7.** Comparison of $^{19}F$ SSNMR spectra of the sample compressed with 250 MPa of pressure acquired 18 months apart. The blue trace (Initial) was acquired shortly after compression in September 2023, while the red trace (18 Months Later) was acquired in March 2025. Crucially, the sample was stored at ambient conditions and remained packed inside the same NMR rotor for the entire 18-month duration. Both NMR spectra were acquired using identical instrumental parameters (a 30 s recycle delay and 128 scans). The MAS frequency was 50 kHz and the magnetic field was 9.4 T. The near-perfect overlap of the NMR spectra and the unchanged relative intensities between the ambient and high-pressure phases provide unambiguous evidence that the high-pressure polymorph is kinetically stable at room temperature after depressurization.

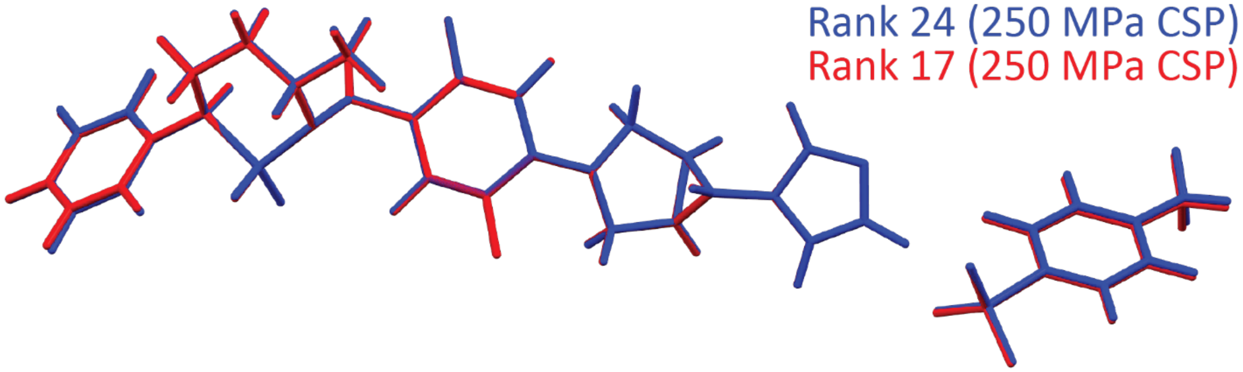


**Figure S8.** Structural overlay of the top two CSP candidates predicted at 250 MPa: Rank 17 (red) and Rank 24 (blue). The overlay reveals a high degree of conformational similarity between the two structures, explaining why they give rise to similar predicted $^{1}$H and $^{19}$F chemical shifts.

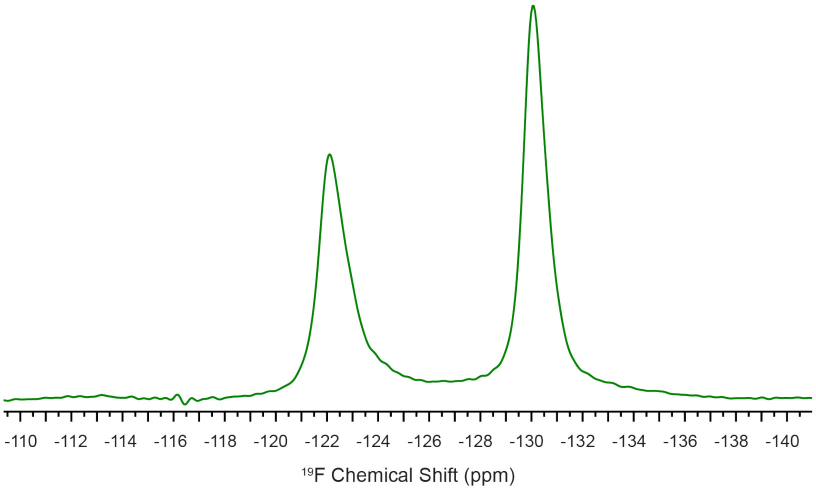


**Figure S9.** $^{19}$F SSNMR spectra of GDC-0022–phosphoric acid cocrystal.

**Table S1.** GIPAW Calculated $^{19}$F Chemical Shifts.

| Sample name | CCDC code | Reported $^{19}$F reference | Reported Exp. $^{19}$F chemical shift (ppm) | Converted Exp. $^{19}$F chemical shift[b] (ppm) | DFT calculated $^{19}$F chemical shielding (ppm) | DFT calculated $^{19}$F chemical shift (ppm) |
|---|---|---|---|---|---|---|
| Levofloxacin Hemihydrate | 865535[11] | TFA aqueous solution[a] | –37.7[12] | –111.25 | 254.84 | –109.47 |
| | | | –39.5[12] | –113.05 | 257.13 | –111.55 |
| Levofloxacin Monohydrate | 983847[13] | TFA aqueous solution[a] | –41.4[12] | –114.95 | 260.84 | –114.91 |
| | | | –42.1[12] | –115.65 | 262.54 | –116.45 |
| Octafluoronaphthalene | 1225837[14] | Teflon $^{19}$F peak = –123.2 ppm | –149.3[15] | –149.3 | 301.37 | –151.67 |
| | | | –154.1[15] | –154.1 | 306.39 | –156.22 |
| | | | –152.8[15] | –152.8 | 300.47 | –150.85 |
| | | | –147.8[15] | –147.8 | 298.28 | –148.87 |
| Flutamide | 1292938[16] | Teflon at −122.0 ppm | –61.5[17] | –62.7 | 201.78 | –61.35 |
| Fluoxetine hydrochloride | 1160514[18] | TFA aqueous solution[a] | 16.8[12] | –56.75 | 201.91 | –61.47 |
| Linezolid form II | 648432[19] | $CFCl_3$ = 0 ppm | –120.85[20] | –120.85 | 265.18 | –118.85 |
| Linezolid form III | 672482[21] | $CFCl_3$ = 0 ppm | –122.4[20] | –122.4 | 268.63 | –121.98 |
| GDC-0022-Phosphoric Acid Cocrystal | 1822441[22] | $CFCl_3$ = 0 ppm | –122.18[c] | –122.18 | 267.29 | –120.76 |
| | | | –130.12[c] | –130.12 | 277.11 | –129.67 |
| GDC-0022-Tosylate Salt | 1822443[22] | $CFCl_3$ = 0 ppm | –122.08[c] | –122.08 | 269.12 | –122.42 |
| | | | –127.22[c] | –127.22 | 274.72 | –127.50 |

[a] "TFA aqueous solution" refers to a 100 µM solution of trifluoroacetic acid in 25 mM sodium phosphate buffer (pH 6.5). Data originally referenced to this solution were converted to the $CFCl_3$ scale by subtracting 73.55 ppm.
[b] All reported and experimental $^{19}$F chemical shifts were calibrated to the standard $CFCl_3$ scale (0 ppm), utilizing secondary references including Teflon at –123.2 ppm and indirect referencing from the $^{1}$H resonance of adamantane at 1.82 ppm.
[c] Experimental $^{19}$F NMR chemical shifts acquired in this work. The corresponding $^{19}$F solid-state NMR spectrum for the GDC-0022–phosphoric acid cocrystal is provided in Figure S9.

**Table S2.** GIPAW Calculated $^{1}H$ Chemical Shifts of Theophylline and Histidine•HCl•$H_2O$

| Sample name | CCDC code | DFT calculated $^{1}H$ chemical shielding (ppm) | Reported Exp. $^{1}H$ chemical shift (ppm)[23,24] | DFT calculated $^{1}H$ chemical shift (ppm) |
|---|---|---|---|---|
| Theophylline Form I | 878132[25] | 27.02 | 3.36 | 3.61 |
| | | 21.68 | 8.61 | 8.27 |
| | | 17.16 | 11.98 | 12.22 |
| Theophylline Monohydrate | 878137[25] | 27.22 | 3.40 | 3.43 |
| | | 22.25 | 7.99 | 7.77 |
| | | 14.66 | 13.82 | 14.40 |
| Theophylline Form II | 878133[25] | 27.02 | 3.31 | 3.61 |
| | | 22.60 | 7.60 | 7.47 |
| | | 14.54 | 14.53 | 14.51 |
| L-Histidine•HCl•$H_2O$ | 1176647[26] | 27.80 | 3.05 | 2.93 |
| | | 27.47 | 3.05 | 3.22 |
| | | 24.47 | 5.35 | 5.84 |
| | | 22.67 | 7.52 | 7.41 |
| | | 21.41 | 8.50 | 8.51 |
| | | 20.90 | 9.22 | 8.95 |
| | | 17.00 | 12.75 | 12.36 |
| | | 11.97 | 17.25 | 16.75 |
| | | **Mean Absolute Error (MAE, ppm)** | | 0.25 |
| | | **Standard deviation of MAE (ppm)** | | 0.172 |
| | | **RMSD (ppm)** | | 0.30 |

Chemical shifts of different theophylline polymorphs were taken from reference 23. The $^{1}H$ NMR spectrum of L-Histidine•HCl•$H_2O$ was measured at 9.4 T with a 50 kHz MAS frequency in our lab, allowing for accurate assignment of the two high frequency $^{1}H$ chemical shifts. However, the chemical shifts of other $^{1}H$ NMR signals were taken from reference 24, which shows the $^{1}H$ solid-state NMR spectrum recorded with a 60 kHz MAS frequency and a 16.4 T magnetic field. The higher magnetic field provides a more resolved $^{1}H$ NMR spectrum. The chemical shifts in reference 24 were corrected by increasing their reported values by 0.5 ppm based upon the comparison of their reported chemical shifts and our measured $^{1}H$ NMR spectra.

**Table S3.** Crystallographic parameters and relative free energies of the 12 candidate structures generated by CSP with 0 MPa of pressure.

| Rank | Space Group | a,b,c (Å) | α,β,γ (°) | $V$ (Å$^3$) | $\rho_{calc}$ (g/cm$^3$) | $Z$ ($Z'$)[a] | $\Delta G$ (kcal/mol)[b] |
|---|---|---|---|---|---|---|---|
| 1 | $P2_12_12_1$ | 6.37, 7.34, 68.89 | 90.0, 90.0, 90.0 | 3222 | 1.385 | 4 (1) | 0.00 |
| 2 | $P2_12_12_1$ | 6.37, 7.35, 68.88 | 90.0, 90.0, 90.0 | 3222 | 1.385 | 4 (1) | 0.02 |
| 3 | $P2_1$ | 6.03, 7.60, 36.53 | 90.0, 89.4, 90.0 | 1675 | 1.332 | 2 (1) | 0.34 |
| 4 | $P2_12_12_1$ | 7.35, 12.63, 68.76 | 90.0, 90.0, 90.0 | 6383 | 1.398 | 8 (2) | 0.51 |
| 5 | $P2_1$ | 15.96, 6.00, 34.24 | 90.0, 101.6, 90.0 | 3214 | 1.389 | 4 (2) | 0.72 |
| 6 | $P2_1$ | 15.96, 6.00, 33.57 | 90.0, 88.1, 90.0 | 3214 | 1.388 | 4 (2) | 0.75 |
| 7 | $P2_1$ | 12.31, 9.74, 13.19 | 90.0, 79.6, 90.0 | 1555 | 1.434 | 2 (1) | 0.94 |
| 8 | $P2_1$ | 7.40, 12.65, 33.85 | 90.0, 85.6, 90.0 | 3162 | 1.411 | 4 (2) | 1.38 |
| 9 | $P2_1$ | 8.04, 5.95, 33.91 | 90.0, 80.9, 90.0 | 1603 | 1.392 | 2 (1) | 1.48 |
| 10 | $P2_1$ | 11.89, 8.14, 32.78 | 90.0, 93.3, 90.0 | 3167 | 1.409 | 4 (2) | 1.80 |
| 11 | $P2_12_12_1$ | 6.77, 17.47, 52.82 | 90.0, 90.0, 90.0 | 6250 | 1.428 | 8 (2) | 1.90 |
| 12 | $P2_1$ | 12.24, 8.03, 32.02 | 90.0, 88.6, 90.0 | 3146 | 1.418 | 4 (2) | 2.18 |

[a] $Z$ denotes the number of formula units per unit cell; $Z'$ denotes the number of formula units in the asymmetric unit.
[b] Relative free energies are calculated with respect to the global minimum (Rank 1).

**Table S4.** Crystallographic parameters and relative free energies of the 24 candidate structures generated by CSP with 250 MPa of applied pressure.

| Rank | Space Group | a,b,c (Å) | α,β,γ (°) | $V$ (Å$^3$) | $\rho_{calc}$ (g/cm$^3$) | $Z$ ($Z'$)[a] | $\Delta G$ (kcal/mol)[b] |
|---|---|---|---|---|---|---|---|
| 1 | $P2_12_12_1$ | 6.27, 7.30, 68.63 | 90.0, 90.0, 90.0 | 3139 | 1.422 | 4 (1) | 0.00 |
| 2 | $P2_1$ | 12.22, 9.70, 13.02 | 90.0, 80.3, 90.0 | 1520 | 1.468 | 2 (1) | 0.16 |
| 3 | $P2_12_12_1$ | 6.29, 7.32, 67.26 | 90.0, 90.0, 90.0 | 3097 | 1.441 | 4 (1) | 0.65 |
| 4 | $P2_1$ | 7.97, 5.90, 33.39 | 90.0, 85.7, 90.0 | 1566 | 1.425 | 2 (1) | 0.75 |
| 5 | $P2_12_12_1$ | 9.49, 16.60, 19.86 | 90.0, 90.0, 90.0 | 3130 | 1.425 | 4 (1) | 0.84 |
| 6 | $P2_12_12_1$ | 6.17, 7.44, 67.69 | 90.0, 90.0, 90.0 | 3106 | 1.436 | 4 (1) | 1.05 |
| 7 | $P2_1$ | 9.09, 6.69, 25.37 | 90.0, 91.6, 90.0 | 1543 | 1.446 | 2 (1) | 1.06 |
| 8 | $P2_1$ | 6.10, 7.77, 33.02 | 90.0, 92.9, 90.0 | 1564 | 1.426 | 2 (1) | 1.31 |
| 9 | $P2_12_12_1$ | 6.14, 7.38, 68.11 | 90.0, 90.0, 90.0 | 3087 | 1.446 | 4 (1) | 1.38 |
| 10 | $P2_12_12_1$ | 9.36, 11.48, 29.74 | 90.0, 90.0, 90.0 | 3195 | 1.397 | 4 (1) | 1.39 |
| 11 | $P2_12_12_1$ | 5.87, 8.06, 67.61 | 90.0, 90.0, 90.0 | 3201 | 1.394 | 4 (1) | 1.43 |
| 12 | $P2_12_12_1$ | 6.07, 7.34, 68.92 | 90.0, 90.0, 90.0 | 3071 | 1.453 | 4 (1) | 1.49 |
| 13 | $P2_1$ | 6.81, 7.16, 31.54 | 90.0, 88.1, 90.0 | 1539 | 1.450 | 2 (1) | 1.50 |
| 14 | $P2_12_12_1$ | 7.30, 8.28, 50.84 | 90.0, 90.0, 90.0 | 3074 | 1.452 | 4 (1) | 1.62 |
| 15 | $P2_12_12_1$ | 7.31, 12.76, 32.97 | 90.0, 90.0, 90.0 | 3076 | 1.451 | 4 (1) | 1.64 |

| Rank | Space Group | a,b,c (Å) | α,β,γ (°) | $V$ (Å$^3$) | $\rho_{calc}$ (g/cm$^3$) | $Z$ ($Z'$)[a] | $\Delta G$ (kcal/mol)[b] |
|---|---|---|---|---|---|---|---|
| 16 | $P2_12_12_1$ | 6.04, 7.76, 65.62 | 90.0, 90.0, 90.0 | 3078 | 1.450 | 4 (1) | 1.78 |
| 17 | $P2_1$ | 6.03, 7.91, 32.84 | 90.0, 94.0, 90.0 | 1562 | 1.429 | 2 (1) | 1.82 |
| 18 | $P2_12_12_1$ | 5.93, 7.92, 66.90 | 90.0, 90.0, 90.0 | 3143 | 1.420 | 4 (1) | 1.83 |
| 19 | $P2_1$ | 6.00, 8.23, 31.64 | 90.0, 83.0, 90.0 | 1550 | 1.439 | 2 (1) | 1.95 |
| 20 | $P2_12_12_1$ | 6.70, 9.95, 45.14 | 90.0, 90.0, 90.0 | 3009 | 1.483 | 4 (1) | 2.17 |
| 21 | $P2_12_12_1$ | 9.45, 12.06, 27.15 | 90.0, 90.0, 90.0 | 3095 | 1.442 | 4 (1) | 2.28 |
| 22 | $P2_12_12_1$ | 5.98, 8.29, 62.14 | 90.0, 90.0, 90.0 | 3080 | 1.449 | 4 (1) | 2.52 |
| 23 | $P2_1$ | 7.38, 11.95, 16.93 | 90.0, 93.9, 90.0 | 1490 | 1.497 | 2 (1) | 2.53 |
| 24 | $P2_12_12_1$ | 6.06, 7.83, 65.71 | 90.0, 90.0, 90.0 | 3118 | 1.431 | 4 (1) | 2.58 |

[a] $Z$ denotes the number of formula units per unit cell; $Z'$ denotes the number of formula units in the asymmetric unit.
[b] Relative free energies are calculated with respect to the global minimum (Rank 1).

**Table S5.** DFT-predicted $^{19}$F isotropic magnetic shieldings and chemical shifts for the 12 candidate structures generated by CSP with 0 MPa of applied pressure.

| Rank | DFT calculated $^{19}$F chemical shielding ($\sigma_{iso}$, ppm) | | DFT calculated $^{19}$F chemical shift ($\delta_{iso}$, ppm) | | RMSD |
|---|---|---|---|---|---|
| 1 | 260.90 | 279.30 | –114.97 | –131.65 | 5.93 |
| 2 | 261.24 | 279.09 | –115.27 | –131.46 | 5.67 |
| **3** | **268.92** | **275.24** | **–122.24** | **–127.97** | **0.54** |
| 4 | 260.15 | 280.58 | –114.29 | –132.81 | 6.78 |
| 4' | 260.76 | 273.03 | –114.84 | –125.97 | 5.20 |
| 5 | 262.68 | 282.97 | –116.58 | –134.98 | 6.73 |
| 5' | 263.10 | 281.95 | –116.96 | –134.06 | 6.04 |
| 6 | 262.83 | 283.00 | –116.72 | –135.01 | 6.69 |
| 6' | 263.10 | 282.12 | –116.96 | –134.21 | 6.13 |
| 7 | 263.66 | 286.00 | –117.47 | –137.73 | 8.11 |
| 8 | 263.55 | 272.50 | –117.37 | –125.49 | 3.55 |
| 8' | 258.37 | 281.69 | –112.67 | –133.82 | 8.13 |
| 9 | 262.43 | 281.03 | –116.35 | –133.22 | 5.87 |
| 10 | 262.81 | 282.52 | –116.70 | –134.57 | 6.44 |
| 10' | 266.55 | 278.44 | –120.09 | –130.87 | 2.94 |
| 11 | 271.00 | 282.98 | –124.12 | –134.99 | 5.68 |
| 11' | 267.96 | 281.87 | –121.37 | –133.98 | 4.81 |
| 12 | 263.64 | 278.28 | –117.45 | –130.73 | 4.11 |
| 12' | 264.57 | 279.54 | –118.29 | –131.87 | 4.24 |
| **XRD** | **269.12** | **274.72** | **–122.42** | **–127.50** | **0.31** |
| | **Experiment** | | **–122.3** | **–127.9** | **-** |

**Table S6.** DFT-predicted $^{1}H$ isotropic magnetic shieldings and chemical shifts for the 12 candidate structures generated by CSP with 0 MPa of pressure.

| Rank | DFT calculated $^{1}H$ chemical shielding ($\sigma_{iso}$, ppm) | | | DFT calculated $^{1}H$ chemical shift ($\delta_{iso}$, ppm) | | | RMSD (ppm) |
|---|---|---|---|---|---|---|---|
| | Ammonium NH | Triazole CH | | Ammonium NH | Triazole CH | | |
| 1 | 9.52 | 18.32 | 17.33 | 18.89 | 11.20 | 12.07 | 2.70 |
| 2 | 10.55 | 19.55 | 18.61 | 17.99 | 10.13 | 10.95 | 1.68 |
| **3** | **12.39** | **21.69** | **20.18** | **16.38** | **8.26** | **9.58** | **0.44** |
| 4 | 12.10 | 20.47 | 19.71 | 16.64 | 9.33 | 9.99 | 0.87 |
| 4' | 12.40 | 20.82 | 20.13 | 16.37 | 9.02 | 9.62 | 0.67 |
| 5 | 12.71 | 20.6 | 19.29 | 16.10 | 9.21 | 10.36 | 1.07 |
| 5' | 12.57 | 20.8 | 19.72 | 16.23 | 9.04 | 9.98 | 0.84 |
| 6 | 12.81 | 20.71 | 19.42 | 16.02 | 9.12 | 10.24 | 1.02 |
| 6' | 12.73 | 21.01 | 19.90 | 16.09 | 8.86 | 9.83 | 0.77 |
| 7 | 13.28 | 20.97 | 19.17 | 15.61 | 8.89 | 10.46 | 1.19 |
| 8 | 13.26 | 21.65 | 20.92 | 15.62 | 8.30 | 8.93 | 0.77 |
| 8' | 12.77 | 21.32 | 20.46 | 16.05 | 8.59 | 9.34 | 0.59 |
| 9 | 12.83 | 21.24 | 19.81 | 16.00 | 8.66 | 9.90 | 0.78 |
| 10 | 13.05 | 21.50 | 19.36 | 15.81 | 8.43 | 10.30 | 0.98 |
| 10' | 13.07 | 21.33 | 19.37 | 15.79 | 8.58 | 10.29 | 1.00 |
| 11 | 11.17 | 21.07 | 17.41 | 17.45 | 8.80 | 12.00 | 1.75 |
| 11' | 11.18 | 21.09 | 17.44 | 17.44 | 8.79 | 11.97 | 1.73 |
| 12 | 13.40 | 21.99 | 19.28 | 15.50 | 8.00 | 10.37 | 1.12 |
| 12' | 12.98 | 21.52 | 19.54 | 15.87 | 8.41 | 10.14 | 0.89 |
| **XRD**[a] | **12.33** | **21.87** | **20.45** | **16.43** | **8.11** | **9.35** | **0.34** |
| | **Experiment** | | | **16.9** | **8.1** | **9.0** | **-** |

[a]XRD refers to the calculation that was performed on the known single-crystal X-ray structure.

**Table S7.** Statistical evaluation of the agreement between experimental and DFT-calculated $^1$H and $^{19}$F chemical shifts for the 12 candidate structures generated by CSP with 0 MPa of applied pressure.

| Structure Candidate | Reduced $\chi^{2\,a}$ | R values$^b$ | Uniform Chi-squared Probability ($P_{\mathrm{UC}}$)$^c$ | Product-Normal Probability ($P_{\mathrm{N}}$)$^c$ |
|---|---|---|---|---|
| **3** | **0.55** | **1.00** | **98.45** | **99.97** |
| 12 | 4.18 | 7.60 | 0.31 | 0.01 |
| 9 | 4.42 | 8.04 | 0.26 | 0.01 |
| 8 | 4.45 | 8.09 | 0.25 | 0.01 |
| 4 | 4.58 | 8.33 | 0.23 | 0.00 |
| 10 | 4.66 | 8.47 | 0.22 | 0.00 |
| 6 | 5.53 | 10.06 | 0.13 | 0.00 |
| 5 | 5.79 | 10.53 | 0.11 | 0.00 |
| 7 | 9.14 | 16.62 | 0.02 | 0.00 |
| 2 | 10.21 | 18.56 | 0.02 | 0.00 |
| 11 | 10.45 | 19.00 | 0.02 | 0.00 |
| 1 | 22.65 | 41.18 | 0.00 | 0.00 |

$^a$ The reduced $\chi^2$ ($\chi^2_{red}$) was calculated using the following equation:

$$\chi^2_{red} = \frac{1}{5}\sum\left(\frac{\delta_{iso}(exp)_j - \delta_{iso}(calc)_j}{RMSD}\right)^2$$

In this equation, the difference is calculated between each of the 5 experimental and calculated isotropic chemical shifts (three $^1$H shifts and two $^{19}$F shifts). RMSD was 0.47 ppm for $^1$H chemical shifts and 2.23 ppm for $^{19}$F chemical shifts. For candidate structures with $Z' = 2$, the reported $\chi^2_{red}$ is the arithmetic mean of the values calculated for the two independent molecules in the asymmetric unit.

$^b$ $R$ values are calculated by dividing $\chi^2_{red}$ of the respective candidate structure by that of the best-fit structure (Structure 3).

$^c$ Uniform Chi-squared ($P_{\mathrm{UC}}$) and Product-Normal ($P_{\mathrm{N}}$) probabilities are expressed as percentages (%) and were calculated utilizing 5 degrees of freedom ($f = 5$) corresponding to the three $^1$H chemical shifts and two $^{19}$F chemical shifts. Calculation of the probabilities was performed using the methods of Mueller.[27]

**Table S8.** DFT-predicted $^{19}F$ isotropic magnetic shieldings and chemical shifts for the 24 candidate structures generated with 250 MPa of applied pressure.

| Rank | DFT calculated $^{19}F$ chemical shielding ($\sigma_{iso}$, ppm) | | DFT calculated $^{19}F$ chemical shift ($\delta_{iso}$, ppm) | | RMSD |
|---|---|---|---|---|---|
| 1 | 261.66 | 278.94 | –115.65 | –131.33 | 6.46 |
| 2 | 262.93 | 284.57 | –116.81 | –136.43 | 6.23 |
| 3 | 267.28 | 274.35 | –120.75 | –127.16 | 4.72 |
| 4 | 260.88 | 278.52 | –114.95 | –130.94 | 6.99 |
| 5 | 267.88 | 276.83 | –121.30 | –129.41 | 3.27 |
| 6 | 258.03 | 274.28 | –112.36 | –127.10 | 9.54 |
| 7 | 267.35 | 279.89 | –120.81 | –132.19 | 2.77 |
| 8 | 262.53 | 283.70 | –116.44 | –135.64 | 6.24 |
| 9 | 256.92 | 276.78 | –111.36 | –129.37 | 9.71 |
| 10 | 263.66 | 274.90 | –117.47 | –127.66 | 6.17 |
| 11 | 266.95 | 278.01 | –120.45 | –130.48 | 3.34 |
| 12 | 258.76 | 277.66 | –113.02 | –130.16 | 8.43 |
| 13 | 254.48 | 282.41 | –109.14 | –134.47 | 11.09 |
| 14 | 267.31 | 277.25 | –120.78 | –129.79 | 3.39 |
| 15 | 260.43 | 266.68 | –114.54 | –120.21 | 11.30 |
| 16 | 262.11 | 277.74 | –116.06 | –130.24 | 6.33 |
| **17** | **268.32** | **279.66** | **–121.69** | **–131.98** | **2.17** |
| 18 | 259.42 | 276.99 | –113.62 | –129.56 | 8.12 |
| 19 | 270.02 | 277.55 | –123.24 | –130.07 | 2.04 |
| 20 | 264.95 | 271.88 | –118.64 | –124.92 | 6.89 |
| 21 | 266.63 | 275.90 | –120.16 | –128.57 | 4.27 |
| 22 | 270.26 | 277.26 | –123.45 | –129.80 | 2.13 |
| 23 | 267.75 | 268.54 | –121.18 | –121.89 | 7.93 |
| **24** | **267.89** | **279.53** | **–121.30** | **–131.86** | **2.46** |
| | **Experiment** | | **–124.7** | **–132.5** | **-** |

**Table S9.** DFT-predicted $^{1}H$ isotropic magnetic shieldings and chemical shifts for the 24 candidate structures generated by CSP with 250 MPa of applied pressure.

| Rank | DFT calculated $^{1}H$ chemical shielding ($\sigma_{iso}$, ppm) | | | DFT calculated $^{1}H$ chemical shift ($\delta_{iso}$, ppm) | | | RMSD (ppm) |
|---|---|---|---|---|---|---|---|
| | Ammonium NH | Triazole CH | | Ammonium NH | Triazole CH | | |
| 1 | 11.29 | 20.31 | 19.42 | 17.34 | 9.47 | 10.24 | 1.04 |
| 2 | 13.11 | 20.69 | 18.97 | 15.75 | 9.14 | 10.64 | 0.61 |
| 3 | 12.14 | 20.88 | 19.40 | 16.60 | 8.97 | 10.26 | 0.58 |
| 4 | 12.22 | 20.89 | 19.34 | 16.53 | 8.96 | 10.31 | 0.57 |
| 5 | 11.34 | 21.53 | 18.91 | 17.30 | 8.40 | 10.69 | 1.04 |
| 6 | 10.99 | 19.30 | 18.20 | 17.60 | 10.35 | 11.31 | 1.69 |
| 7 | 11.95 | 20.46 | 19.09 | 16.77 | 9.34 | 10.53 | 0.82 |
| 8 | 13.22 | 21.17 | 20.33 | 15.66 | 8.72 | 9.45 | 0.17 |
| 9 | 11.43 | 19.44 | 18.43 | 17.22 | 10.23 | 11.11 | 1.46 |
| 10 | 12.29 | 19.25 | 17.83 | 16.47 | 10.39 | 11.63 | 1.55 |
| 11 | 11.58 | 19.88 | 18.53 | 17.09 | 9.84 | 11.02 | 1.26 |
| 12 | 11.82 | 19.78 | 18.82 | 16.88 | 9.93 | 10.77 | 1.13 |
| 13 | 13.11 | 21.03 | 20.40 | 15.75 | 8.84 | 9.39 | 0.20 |
| 14 | 11.97 | 22.03 | 20.58 | 16.75 | 7.97 | 9.23 | 0.72 |
| 15 | 12.43 | 21.50 | 18.51 | 16.35 | 8.43 | 11.04 | 0.85 |
| 16 | 12.54 | 20.33 | 20.04 | 16.25 | 9.45 | 9.70 | 0.51 |
| **17** | **12.91** | **20.75** | **20.34** | **15.93** | **9.08** | **9.44** | **0.28** |
| 18 | 10.55 | 19.18 | 17.62 | 17.99 | 10.45 | 11.82 | 2.03 |
| 19 | 11.85 | 20.94 | 18.69 | 16.85 | 8.92 | 10.88 | 0.92 |
| 20 | 10.81 | 19.39 | 19.20 | 17.76 | 10.27 | 10.44 | 1.51 |
| 21 | 11.93 | 20.19 | 19.78 | 16.78 | 9.57 | 9.93 | 0.77 |
| 22 | 11.72 | 20.79 | 18.72 | 16.97 | 9.05 | 10.86 | 0.97 |
| 23 | 13.27 | 18.10 | 13.09 | 15.61 | 11.40 | 15.77 | 4.21 |
| **24** | **13.00** | **20.80** | **20.25** | **15.85** | **9.04** | **9.52** | **0.23** |
| | **Experiment** | | | **15.8** | **8.6** | **9.6** | **-** |

**Table S10.** Statistical evaluation of the agreement between experimental and DFT-calculated [1]H and [19]F chemical shifts for the 23 candidate structures generated with CSP under 250 MPa of applied pressure.

| Structure Candidate | Reduced $\chi^{2\,a}$ | R values$^b$ | Uniform Chi-squared Probability ($P_{UC}$)$^c$ | Product-Normal Probability ($P_N$)$^c$ |
|---|---|---|---|---|
| **17**$^d$ | **0.59** | **1.00** | **88.85** | **96.10** |
| 14 | 2.33 | 3.95 | 2.07 | 1.24 |
| 7 | 2.46 | 4.17 | 1.76 | 0.90 |
| 19 | 2.64 | 4.48 | 1.42 | 0.57 |
| 3 | 2.72 | 4.61 | 1.30 | 0.47 |
| 22 | 2.91 | 4.93 | 1.06 | 0.29 |
| 21 | 3.07 | 5.20 | 0.90 | 0.20 |
| 8 | 3.21 | 5.44 | 0.79 | 0.14 |
| 5 | 3.81 | 6.46 | 0.46 | 0.03 |
| 16 | 3.92 | 6.64 | 0.42 | 0.02 |
| 2 | 4.14 | 7.02 | 0.36 | 0.01 |
| 4 | 4.82 | 8.17 | 0.22 | 0.00 |
| 11 | 5.19 | 8.80 | 0.17 | 0.00 |
| 1 | 6.28 | 10.64 | 0.09 | 0.00 |
| 12 | 9.21 | 15.61 | 0.03 | 0.00 |
| 10 | 9.55 | 16.19 | 0.02 | 0.00 |
| 13 | 10.00 | 16.95 | 0.02 | 0.00 |
| 20 | 10.01 | 16.97 | 0.02 | 0.00 |
| 15 | 12.25 | 20.76 | 0.01 | 0.00 |
| 9 | 13.34 | 22.61 | 0.01 | 0.00 |
| 6 | 15.11 | 25.61 | 0.01 | 0.00 |
| 18 | 16.51 | 27.98 | 0.00 | 0.00 |
| 3 | 53.32 | 90.37 | 0.00 | 0.00 |

$^a$ The reduced $\chi^2$ ($\chi^2_{red}$) was calculated as described above in Table S7.

$^b$ $R$ values are calculated by dividing $\chi^2_{red}$ of the respective candidate structure by that of the best-fit structure (structure 17).

$^c$ Uniform Chi-squared ($P_{UC}$) and Product-Normal ($P_N$) probabilities are expressed as percentages (%) and were calculated utilizing 5 degrees of freedom ($f = 5$) corresponding to the three [1]H chemical shifts and two [19]F chemical shifts. Calculation of the probabilities was performed using the methods of Mueller.[27]

$^d$ Structure 24 ($\chi^2_{red}$ = 0.63) was excluded from the statistical probability normalization to prevent probability dilution. Structure 24 is a redundant model with structure 17, as described in the main text.

## Supplementary Information References:


1. Harris, R. K.; Becker, E. D.; Cabral de Menezes, S. M.; Goodfellow, R.; Granger, P., NMR Nomenclature: Nuclear Spin Properties and Conventions for Chemical Shifts: IUPAC Recommendations 2001. *Solid State Nucl Magn Reson* **2002,** *22* (4), 458-483.
2. Thakur, R. S.; Kurur, N. D.; Madhu, P. K., Swept-frequency two-pulse phase modulation for heteronuclear dipolar decoupling in solid-state NMR. *Chem. Phys. Lett.* **2006,** *426* (4), 459-463.
3. Metz, G.; Wu, X. L.; Smith, S. O., Ramped-Amplitude Cross Polarization in Magic-Angle-Spinning NMR. *J. Magn. Reson.* **1994,** *110* (2), 219-227.
4. Peersen, O. B.; Wu, X. L.; Kustanovich, I.; Smith, S. O., Variable-Amplitude Cross-Polarization MAS NMR. *J. Magn. Reson.* **1993,** *104* (3), 334-339.
5. Sakellariou, D.; Lesage, A.; Hodgkinson, P.; Emsley, L., Homonuclear dipolar decoupling in solid-state NMR using continuous phase modulation. *Chem. Phys. Lett.* **2000,** *319* (3), 253-260.
6. Fung, B. M.; Khitrin, A. K.; Ermolaev, K., An improved broadband decoupling sequence for liquid crystals and solids. *J Magn Reson* **2000,** *142* (1), 97-101.
7. Feike, M.; Demco, D. E.; Graf, R.; Gottwald, J.; Hafner, S.; Spiess, H. W., Broadband Multiple-Quantum NMR Spectroscopy. *J. Magn. Reson., Ser. A* **1996,** *122* (2), 214-221.
8. Wijesekara, A. V.; Venkatesh, A.; Lampkin, B. J.; VanVeller, B.; Lubach, J. W.; Nagapudi, K.; Hung, I.; Gor'kov, P. L.; Gan, Z.; Rossini, A. J., Fast Acquisition of Proton-Detected HETCOR Solid-State NMR Spectra of Quadrupolar Nuclei and Rapid Measurement of NH Bond Lengths by Frequency Selective HMQC and RESPDOR Pulse Sequences. *Chem. Eur. J.* **2020,** *26* (35), 7881-7888.
9. Hodgkinson, P., NMR crystallography of molecular organics. *Prog. Nucl. Magn. Reson. Spectrosc* **2020,** *118-119*, 10-53.
10. Dumon, A. S.; Rzepa, H. S.; Alamillo-Ferrer, C.; Bures, J.; Procter, R.; Sheppard, T. D.; Whiting, A., A computational tool to accurately and quickly predict 19F NMR chemical shifts of molecules with fluorine–carbon and fluorine–boron bonds. *Phys. Chem. Chem. Phys.* **2022,** *24* (34), 20409-20425.
11. Gorman, E. M.; Samas, B.; Munson, E. J., Understanding the dehydration of levofloxacin hemihydrate. *J. Pharm. Sci.* **2012,** *101* (9), 3319-3330.
12. Quinn, C. M.; Zadorozhnyi, R.; Struppe, J.; Sergeyev, I. V.; Gronenborn, A. M.; Polenova, T., Fast 19F Magic-Angle Spinning Nuclear Magnetic Resonance for the Structural Characterization of Active Pharmaceutical Ingredients in Blockbuster Drugs. *Anal. Chem.* **2021,** ***93*** (38), 13029-13037.
13. Singh, S. S.; Thakur, T. S., New crystalline salt forms of levofloxacin: conformational analysis and attempts towards the crystal structure prediction of the anhydrous form. *CrystEngComm* **2014,** *16* (20), 4215-4230.
14. Akhmed, N., Study of Crystalline-Structure of Octafluoronaphthalene. 1973; Vol. 14, pp 573-574.
15. Robbins, A. J.; Ng, W. T. K.; Jochym, D.; Keal, T. W.; Clark, S. J.; Tozer, D. J.; Hodgkinson, P., Combining insights from solid-state NMR and first principles calculation: applications to the 19F NMR of octafluoronaphthalene. *Phys. Chem. Chem. Phys.* **2007,** *9* (19), 2389-2396.
16. Cense, J.; Agafonov, V.; Ceolin, R.; Ladure, P.; Rodier, N., Crystal and molecular structure analysis of flutamide. Bifurcated helicoidal CH··· O hydrogen bonds. *Struct. Chem.* **1994,** *5* (2), 79-84.
17. Phyo, P.; Xu, W.; Frank, D.; Li, T.; Su, Y., Probing Molecular Packing of Drug Substances in Nanometer Domains in Pharmaceutical Formulations Using 19F Magic Angle Spinning NMR. *J. Phys. Chem. C* **2022,** *126* (29), 12025-12037.
18. Robertson, D. W.; Jones, N. D.; Swartzendruber, J. K.; Yang, K. S.; Wong, D. T., Molecular structure of fluoxetine hydrochloride, a highly selective serotonin-uptake inhibitor. *J. Med. Chem.* **1988,** *31* (1), 185-189.
19. Maccaroni, E.; Alberti, E.; Malpezzi, L.; Masciocchi, N.; Vladiskovic, C., Polymorphism of linezolid: A combined single-crystal, powder diffraction and NMR study. *Int. J. Pharm.* **2008,** *351* (1-2), 144-151.

20. Wielgus, E.; Paluch, P.; Frelek, J.; Szczepek, W. J.; Potrzebowski, M. J., Full characterization of linezolid and its synthetic precursors by solid-state nuclear magnetic resonance spectroscopy and mass spectrometry. *J. Pharm. Sci.* **2015,** *104* (11), 3883-3892.
21. TANAKA, R.; HIRAYAMA, N., Crystal structure of linezolid. *Anal. Sci.: X-Ray Struct. Anal. Online* **2008,** *24*, x43-x44.
22. Zhao, L.; Hanrahan, M. P.; Chakravarty, P.; DiPasquale, A. G.; Sirois, L. E.; Nagapudi, K.; Lubach, J. W.; Rossini, A. J., Characterization of Pharmaceutical Cocrystals and Salts by Dynamic Nuclear Polarization-Enhanced Solid-State NMR Spectroscopy. *Cryst. Growth Des.* **2018,** *18* (4), 2588-2601.
23. Hirsh, D. A.; Wijesekara, A. V.; Carnahan, S. L.; Hung, I.; Lubach, J. W.; Nagapudi, K.; Rossini, A. J., Rapid Characterization of Formulated Pharmaceuticals Using Fast MAS 1H Solid-State NMR Spectroscopy. *Mol. Pharm.* **2019,** *16* (7), 3121-3132.
24. Mithu, V. S.; Tan, K. O.; Madhu, P. K., Selective inversion of 1H resonances in solid-state nuclear magnetic resonance: Use of double-DANTE pulse sequence. *J. Magn. Reson.* **2013,** *237*, 11-16.
25. Fucke, K.; McIntyre, G. J.; Wilkinson, C.; Henry, M.; Howard, J. A.; Steed, J. W., New insights into an old molecule: interaction energies of theophylline crystal forms. *Cryst. Growth Des.* **2012,** *12* (3), 1395-1401.
26. Oda, K.; Koyama, H., A refinement of the crystal structure of histidine hydrochloride monohydrate. *Acta Crystallogr. B* **1972,** *28* (2), 639-642.
27. Mueller, L. J., Uniform chi-squared model probabilities in NMR crystallography. *Faraday Discuss.* **2025,** *255*, 203-221.
